# Hydration and Structural Adaptations of the Human CYP1A1, CYP1A2, and CYP1B1 Active Sites by Molecular Dynamics Simulations

**DOI:** 10.3390/ijms241411481

**Published:** 2023-07-14

**Authors:** Zbigniew Dutkiewicz, Renata Mikstacka

**Affiliations:** 1 Department of Chemical Technology of Drugs, Poznan University of Medical Sciences, Grunwaldzka 6, 60-780 Poznań, Poland; 2 Department of Inorganic and Analytical Chemistry, Nicolaus Copernicus University, Collegium Medicum, Dr. A. Jurasza 2, 85-089 Bydgoszcz, Poland

**Keywords:** cytochrome P450, CYP1A1, CYP1A2, CYP1B1, molecular docking, molecular dynamics simulation studies, polymethoxy-trans-stilbenes

## Abstract

Cytochromes CYP1A1, CYP1A2, and CYP1B1, the members of the cytochrome P450 family 1, catalyze the metabolism of endogenous compounds, drugs, and non-drug xenobiotics which include substances involved in the process of carcinogenesis, cancer chemoprevention, and therapy. In the present study, the interactions of three selected polymethoxy-trans-stilbenes, analogs of a bioactive polyphenol trans-resveratrol (3,5,4′-trihydroxy-trans-stilbene) with the binding sites of CYP1 isozymes were investigated with molecular dynamics (MD) simulations. The most pronounced structural changes in the CYP1 binding sites were observed in two substrate recognition sites (SRS): SRS2 (helix F) and SRS3 (helix G). MD simulations show that the number and position of water molecules occurring in CYP1 APO and in the structures complexed with ligands are diverse. The presence of water in binding sites results in the formation of water–protein, water–ligand, and bridging ligand–water–protein hydrogen bonds. Analysis of the solvent and substrate channels opening during the MD simulation showed significant differences between cytochromes in relation to the solvent channel and the substrate channels 2c, 2ac, and 2f. The results of this investigation lead to a deeper understanding of the molecular processes that occur in the CYP1 binding sites and may be useful for further molecular studies of CYP1 functions.

## 1. Introduction

Cytochromes P450 are a superfamily of hemoproteins functioning as oxidoreductases. Cytochrome P450 family 1 (CYP1) consists of the enzymes CYP1A1, CYP1A2, and CYP1B1, differing in structure and substrate specificity. The structures and functions of CYP1A1 and CYP1B1 have been widely investigated due to the role these enzymes play in the chemoprevention and therapy of human diseases [1]. CYP1s are responsible for the detoxifying metabolism of xenobiotics that, in the case of procarcinogens, may lead to the generation of carcinogenic products [2,3].

Ligands of CYP1 isozymes include endo- and exogenous substrates, among others: biologically active flavonoids, coumarins, and stilbenoids [4]. CYP1 isozymes, specifically CYP1A2 present in the liver, are mainly responsible for drug metabolism [5]. CYP1B1 catalyzes the 4-hydroxylation of 17β-estradiol to highly carcinogenic products [6]. Hence, CYP1B1 and CYP1A1 are treated as potential targets of anticancer treatment [1]. CYP1A1 and CYP1B1 are mainly present in extrahepatic tissues and are overexpressed in tumors. This feature can be used in cancer therapy with prodrugs activated by CYP1A1 and CYP1B1. On the other hand, natural compounds and their analogs, as inhibitors of CYP1s’ activity, are supposed to be chemopreventive agents in cardiovascular and degenerative diseases [7].

The investigation of conformational changes in CYP1 isozymes using a series of compounds with similar structures allows the identification of the crucial substrate–protein interactions responsible for the function and substrate specificity of isozymes. Stilbenoids are a class of natural and synthetic derivatives of trans-stilbene showing various biological effects studied extensively in cells in vitro and animals in vivo [8]. The bioactivities of trans-stilbenes are supposed to be promising in cardioprotection, neuroprotection, anti-cancer prevention, and therapy.

Computational methods are used to determine enzymes’ molecular structures and characterize the interactions between substrates and enzyme binding sites. Molecular docking and molecular dynamics simulations concerning enzyme substrates or inhibitors are essential tools for drug design and development. Moreover, they allow the prediction of xenobiotic metabolism and drug–drug interactions [4,9]. Since the determination of the crystal structures of CYP1 family members [10,11,12], the substrate specificity of isozymes has been extensively investigated with the use of in silico modeling [13]. First, crystal structures of CYP1 isozymes were determined with α-naphthoflavone as a ligand. Recently, more crystal structures have been deposited in the Protein Data Bank for CYP1A1 with bergamottin, erlotinib [14], Pim kinase inhibitor GDC-0339 [15], and duocarmycin agents [16], and for CYP1B1 with inhibitors having azide groups and estradiol [17,18] as substrates.

Molecular docking and molecular dynamics simulations allow us to explain the functions of enzymes, the role of access channels, and the flexibility of both the binding site of the enzyme and the more distant parts of the protein, which influence the substrate specificity. Interactions of substrates in the binding sites of CYP1 family members were explored with the use of several ligands: series of coumarin derivatives [19], trans-stilbene derivatives [20,21,22,23,24], flavonoids and α-naphthoflavone derivatives [25,26,27,28], and a series of eight compounds—mostly therapeutic agents differing in the specificity toward CYP1s [29]. The selective activity of inhibitors toward CYP1 isoforms is currently being studied with the use of molecular dynamics simulations. These studies shed light on the conformational changes within the segments of the proteins and essential amino acids residues participating in the substrate binding in the catalytic sites of the enzyme [30], and equally important changes in the tunnels which enable the entrance and exit of ligands [31]. On the other hand, this computational technique used in comparative proteomics studies of CYP1s demonstrates the structural proteins’ features, which can determine their substrate specificity [32].

In the last decades, the effect of water molecules on the stability and function of biomolecules has been widely investigated using experimental and computational methods [33]. Water molecules play an important role in ligand–enzyme interactions: mobile water molecules and stable water molecules with long resistance times in the ligand binding pocket are involved in ligand–protein interactions. The function of water in the ligand binding depends on the location: interfacial water forms clusters and networks bridging between the enzyme and its ligand by hydrogen bond formation, whereas water on the surface of the protein may be conserved or displaced during ligand binding. The thermodynamic effect of water replacement by a ligand influences its affinity for an enzyme. Other categories of water molecules comprise buried water, with a long residence time, and bulk water, that participates in cooperative exchange with bound water [34]. MD simulations enable the screening of water mobility and displacements by ligand molecules. Changes in the hydration network in the enzyme cavity are supposed to influence ligand affinity for the binding site. Interfacial water molecules may directly participate in the ligand binding, or a ligand replaces them in the enzyme cavity. Molecular dynamics simulations allow the movements of water molecules to be followed and reciprocal interactions between a ligand, water molecules, and a target protein. There are only a few reports on water molecules’ role in the ligand binding to CYP1 isoforms [19,35,36]. Failure to include water molecules in CYP1 docking is a potential source of error in estimating ligand affinity by scoring functions. Better recognition and understanding of water participation in the ligand–target interactions will facilitate research leading to the development of new effective drugs [37].

At the molecular level, the dependence of their inhibitory activity on the structure of trans-stilbene derivatives has been analyzed [20,21,22]. However, attempts to explain the selectivity of CYP1 isozymes inhibitors have not been entirely satisfactory. In the present study, we employed molecular dynamics simulations to explore the role of water molecules in binding trans-stilbene methoxy derivatives docked to CYP1A1, CYP1A2, and CYP1B1 binding sites. For this purpose, three polymethoxy-trans-stilbenes differing in number and position of methoxy groups were selected as CYP1 ligands. In addition, structural changes induced by trans-stilbene derivatives in the active centers were investigated, with a special focus on solvent and substrate channels. 

## 2. Results and Discussion

**Scheme 1:**
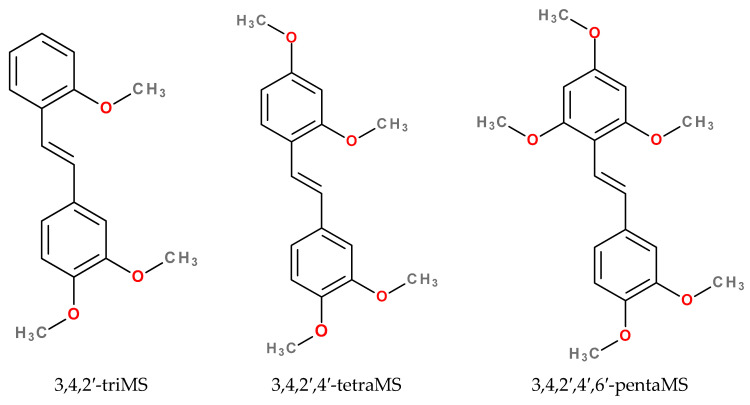
Structures of studied polymethoxy-trans-stilbenes.

Three methoxy-trans-stilbene derivatives (Scheme 1), 3,4,2′-trimethoxy-trans-stilbene (3,4,2′-triMS), 3,4,2′,4′-tetramethoxy-trans-stilbene (3,4,2′,4′-tetraMS), and 3,4,2′,4′,6′-pentamethoxy-trans-stilbene (3,4,2′,4′,6′-pentaMS), were selected from a series of polymethoxy-trans-stilbenes previously studied as inhibitors of the catalytic activity of the CYP1 isoforms (Table 1): CYP1A1, CYP1A2, and CYP1B1 [22]. In the present study, changes in CYP1 molecular structures resulting from ligand binding and enzyme accommodation were investigated with MD simulations. 

The interactions of the tested ligands and binding sites of CYP1s with water molecules were analyzed within 300 ns of MD simulations (400 ns for CYP1A2 APO). During this time, the structures of CYP1A1, CYP1A2, and CYP1B1 bound with the ligands stabilized (Supplementary Materials, Figures S1–S3), indicating the relative flexibility of these proteins.

### 2.1. Structural Changes in Substrate Recognition Sites

The binding of the studied polymethoxy-trans-stilbenes in the catalytic sites of CYP1A1, CYP1A2, and CYP1B1 was primarily by means of hydrophobic interactions as was reported earlier for other substrates/inhibitors [20,21,22]. To a lesser extent, the ligand binding is determined by hydrogen bonds formed by methoxy groups of a ligand in the enzyme cavities. The amino acid residues interacting with the studied ligands via methoxy substituents are included in Table 2. The occupancy, expressed as the percent of the time when an H-bond was observed within the last ten nanoseconds of the MD simulations, gives an idea of the stability of these interactions. The H-bond occupancies for most of the studied CYP1 complexes were negligibly small (occupancy < 0.5%). The exceptions were the CYP1A1-3,4,2′,4′,6′-pentaMS and CYP1A2–3,4,2′-triMS complexes, in which the protein–ligand hydrogen bond was maintained for 10.74% and 27.32% of the simulation time, respectively (Table 2).

**Figure 1:**
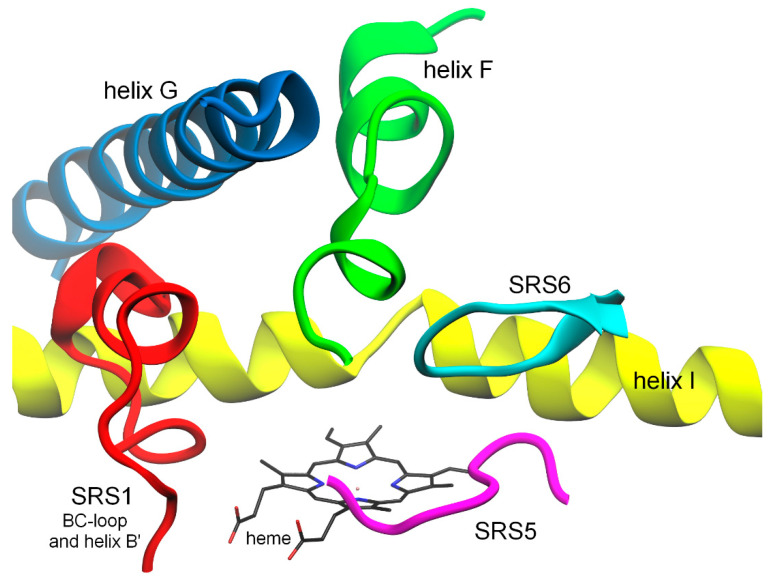
Structural elements surrounding the binding site in CYP1 cytochromes, using CYP1A1 as an example: BC-loop and helix B’ (including SRS1)—red, helix F (including SRS2)—green, helix G (including SRS3)—blue, helix I (including SRS4)—yellow, SRS5—magenta, and SRS6—cyan. Heme is represented as a stick model in black.

Changes in the enzyme’s structure were analyzed by the superimposition of its crystallographic structure on the protein complexed with a ligand. This method visualized not only the change itself, but also its direction, and localized the changes within secondary and tertiary structures (Supplementary Materials, Table S1). Structural elements surrounding the binding sites, including substrate recognition sites (SRSs), in CYP1s, helix B’ or BC-loop (SRS1), and helices F (SRS2), G (SRS3), and I (SRS4), as well as loops where SRS5 and SRS6 are located, are shown in Figure 1. The RMSD calculations within SRSs (Supplementary Material, Tables S2–S4) confirmed the observations from the superimposition of the protein structures.

The tested methoxy-trans-stilbenes induced structural deformations of SRSs (Supplementary Material, Tables S1–S4) indicating the conformational flexibility of the studied proteins. Both methods used, RMSD analyses and superimposition, recognized the SRS2 region as undergoing the biggest structural changes caused by the ligand bound with the enzymes. The CYP1A1 APO structure compared to the X-ray structure is also not much different. The most pronounced change was observed in the region close to Gln212-Phe224, where the F helix turns away from the I helix, and in the SRS4 within Val311-Thr321. A similar effect was observed in the CYP1A2 APO structure, where the distance of the F helix to helix I increased due to the distortion in the F helix (Ser216-Glu228). A different effect was observed in the CYP1B1 APO structure, where the C-terminal end of helix F had shifted towards helix G. The displacement of the F helix in relation to the I helix was also visible for all CYP1 enzymes complexed with the ligands (Supplementary Materials, Table S1). In the CYP1A1 and CYP1A2 complexes, there are also noticeable deformations within SRS1 (BC-loop and B’ helix). A common change in the G helix, seen in the complexes, is moving its N-terminal end away from the F helix. On the other hand, helix I undergoes only slight deformations, usually in the vicinity of heme. SRS5 and SRS6 are the least affected by binding molecules; moreover, SRS5, for all ligands complexed with CYP1A1, CYP1A2, and CYP1B1, coincides with the corresponding fragments of X-ray protein structures.

It is worth noting that the most selective inhibitor, 3,4,2′-triMS, did not cause specific structural changes in the CYP1B1 cavity in any of the SRSs analyzed, which could be responsible for the strong inhibitory activity of the compound. Moreover, the MD simulation confirmed that this compound did not form protein–ligand hydrogen bonds in the CYP1B1 binding site.

Structural rearrangements within the binding site lead to a better accommodation of the ligand in the enzyme cavity. The calculations of Rg (radius of gyration) values that provide information for the binding site’s compactness indicate the effect of ligands on the increase in the size of the binding center. The Rg values calculated for binding site amino acids are given in Table 3. It can be seen that the most pronounced changes in the binding site size occurred in CYP1A2. In turn, among the studied ligands, 3,4,2’,4’,6’-pentamethoxy-trans-stilbene had the greatest impact on the size of the cytochromes’ binding site. It induces changes in Rg for the CYP1A1, CYP1A2, and CYP1B1 binding sites of +7%, +13%, and +8%, respectively.

The root mean square fluctuation (RMSF) of the CYP1 structures, APO, and complexes, presented maxima mainly in loop regions: β1-turn, B’C-loop (SRS1), CD-loop, DE-loop, FG-loop, GH-loop, HI-loop, and loops between β3-1 and SRS6 in CYP1A1/2 or between β3-1 and β4-1 in CYP1B1 (Supplementary Materials, Figures S7–S9).

Considering the changes in the RMSF values for loops in the APO structures and complexes (Supplementary Materials, Figures S7–S9), stabilization of some loops by binding ligands can be seen, for example, the FG-loop in CYP1A2 and CYP1B1 (stabilizing effect most pronounced), the GH-loop in CYP1A2, and the HI-loop in CYP1A1 and CYP1B1. On the other hand, the GH-loop in CYP1B1, HI-loop in CYP1A1, and loop between β3-1 and SRS6 in CYP1A1 show greater fluctuations than the same structural elements in the corresponding ligand-free forms. Variations in the RMSF values at substrate recognition sites, SRSs, can be related to channel opening in proteins and their complexes, as discussed later in this paper.

### 2.2. Hydration of CYP1 Structures in APO Forms and as Complexes with the Polymethoxy-trans-Stilbenes

Water molecules are supposed to play an important role in the interaction of ligands in the binding sites of enzymes; however, in most studies, the presence of water molecules in the binding cavity is omitted. Recently, in the study of interactions of 3-phenylcoumarin derivatives with CYP1 isoforms, the hydrogen bonds via water molecules were shown with MD simulations [19]. On the other hand, a mobile network of water molecules in the binding pocket can also destabilize the ligand binding. Among the CYP1 isozymes, more water molecules were found in CYP1A1 than in CYP1A2 and CYP1B1, according to the binding site volume [19]. The least number of water molecules occurred in CYP1A2, and this isoform was the most efficient in 7-hydroxylation of 3-phenylcoumarin derivatives; in this case, an excess of water molecules did not destabilize the substrate binding [19]. A network of water molecules has been added to calculate the CYP1B1 binding site volume [18].

In the present MD simulations, water molecules were observed either in the binding site of the studied enzymes complexed with ligands or APO forms. Water molecules were displaced by ligands; however, they were still in the enzyme cavity. During the simulation, the average number of water molecules in a ligand’s vicinity was different depending on the ligand and the CYP1 isoform (Table 4). For a given cytochrome, the largest number of water molecules was usually located near the largest ligand, 3,4,2′,4′,6′-pentaMS (within 5.0 angstrom). The lowest average number of water molecules near the ligand was recorded in the complexes of 3,4,2′-triMS with CYP1B1 and 3,4,2′,4′-tetraMS with CYP1A2 (Table 4).

Hydrogen bonds between the studied methoxy-trans-stilbenes and amino acid residues in the active sites of CYP1 isozymes are not formed or are formed rarely (Table 2). On the other hand, water molecules are hydrogen-bonded to the amino acids of the active site, acting as hydrogen bond acceptors or donors. Furthermore, clusters of water molecules can be formed near ligands and they also can bind to the ligand or amino acid residues with hydrogen bonds. However, ‘bridging’ hydrogen bonds, ligand–water molecule–binding site, seem not to play a significant role in ligand–enzyme binding, because these interactions usually last for a short time (Table 5).

#### 2.2.1. Hydration of the Crystal Structures of CYP1A1, CYP1A2, and CYP1B1

CYP1A1’s crystal structure with α-naphthoflavone (ANF) (PDB ID: 4I8V) as a ligand contains water molecules participating in the hydrogen bonds between some residues, but they do not form hydrogen bonds with ANF [12].

In CYP1A2’s crystal structure (PDB ID: 2HI4), one water molecule exists, forming hydrogen bonds with the oxygen atom of the carbonyl group in ANF and the oxygen atom of the carbonyl group in Gly316. In the studies of Watanabe et al. [38], the initial structure of CYP1A2 did not contain any water molecules. They appeared in the active site during MD simulations. The occurrence of water molecules in the CYP1A2 binding site complexed with 7-ethoxyresorufin was demonstrated with the use of long MD simulation (200 ns). Water molecules participated in the bridging hydrogen bond formation between 7-ethoxyresorufin and the residues in the binding site. Another hydrogen bond network was observed during MD simulations of CYP1A2 with α-naphthoflavone (ANF). Water molecules seem not to be necessary for ANF recognition [38].

Substrate selectivity of CYP1A2 is involved with a small volume and relatively less malleable properties. Under high pressure, water molecules are put out of the binding cavity. One should remember that local structural changes close to the heme might, at least to a certain extent, also depend on global structural changes [35].

The crystal structure of CYP1B1 with ANF as a ligand (PDB ID: 3PM0) contains water molecules, but they do not participate in the binding of the ligand to the CYP1B1 active site [11].

#### 2.2.2. Hydration of CYP1A1 Binding Site

In CYP1A1 structures, the SRS1 region and the area between the BC-loop and helix I are strongly hydrated. In the APO form of CYP1A1 and complexes of CYP1A1, stable hydrogen bonds are formed by water with the amino acids of SRS1: Arg106 (APO and all complexes), Pro107 (APO), Asp108 (APO and all complexes), Tyr110 (APO and all complexes), Thr111 (APO, 3,4,2′,4′-tetraMS, 3,4,2′,4′,6′-pentaMS), Phe112 (APO), Thr113 (APO and all complexes), Leu114 (3,4,2′-triMS, 3,4,2′,4′-tetraMS), Ile115 (APO, 3,4,2′,4′-tetraMS and 3,4,2′,4′,6′-pentaMS), Ser116 (APO, 3,4,2′,4′-tetraMS and 3,4,2′,4′,6′-pentaMS), Asn117 (APO and all complexes), Gly118 (APO, 3,4,2′,4′,6′-pentaMS), Gly119 (Apo, 3,4,2′-triMS), Ser120 (APO), Met121 (APO, 3,4,2′-triMS), Ser122 (APO, 3,4,2′,4′,6′-pentaMS), and Phe123 (APO and all complexes). Among the amino acids of helix I, Asn309 (APO and all complexes), Asp313 (APO and all complexes), and Gly316 (APO, 3,4,2′,4′-tetraMS, 3,4,2′,4′,6′-pentaMS) are involved in forming hydrogen bonds with water.

In all of the described CYP1A1 structures, water molecules are also present between the B’ and G helices. Asp253 and Glu256 (G helix) form a hydrogen bond with water in the APO form and all tested complexes.

The area supposed to be a solvent tunnel between the F and I helices and SRS6, in all CYP1A1 structures, is hydrated, but most strongly in the complex with 3,4,2′,4′,6′-pentaMS. The hydration of this site decreases in the order 3,4,2’,4’,6’-pentaMS > 3,4,2’,4’-tetraMS > 3,4,2’-triMS > APO. In complex with 3,4,2′,4′,6′-pentaMS, a branched chain of water molecules can be observed, which form H-bonds with Gly316, Asp320 and Thr323 (helix I), Asn222, Glu226, Val227 in helix F, and Leu496 in the β4 loop (SRS6). In slightly less hydrated complexes with 3,4,2′,4′-tetraMS and 3,4,2′-triMS, the water molecules present between helices F and I and the β4 loop are hydrogen bonded to Asp320, Asn223, Glu226, and Val227 (helix F).

In APO form, the chain of water molecules enters the binding site from the solvent channel and connects to the chain of waters entering from the opposite side along helix I (channel 2c). In ligand-free form, the water molecules form hydrogen bonds with Asp320 and Thr321 (helix I) and Thr497 (SRS6). Tables S5 and S6 (Supplementary Materials) provide detailed information on the water–amino acid hydrogen bonds at the CYP1A1 binding site.

**Figure 2:**
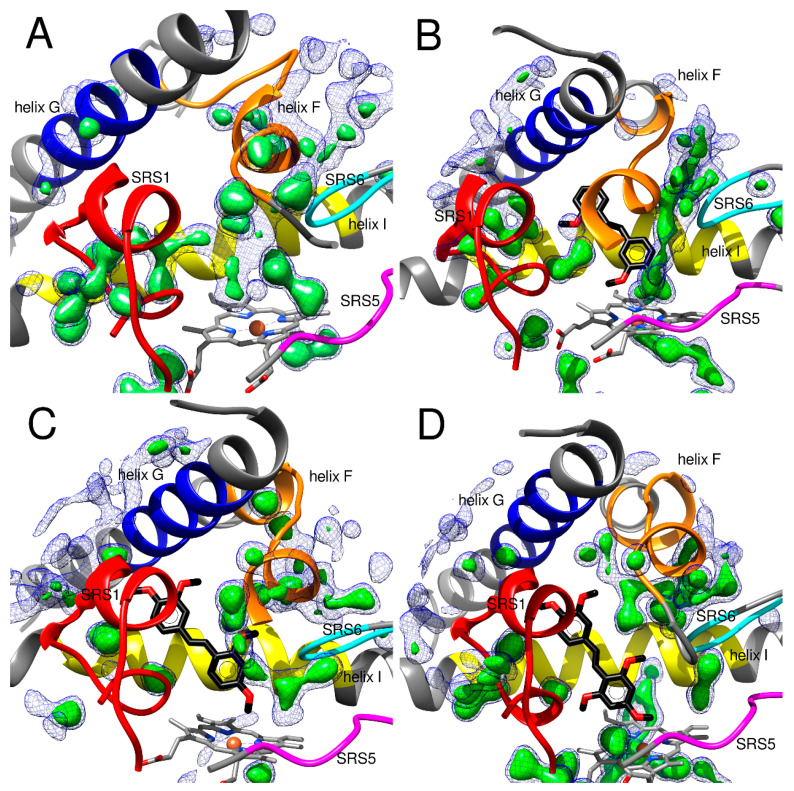
Volumetric maps of water molecule occupancy in the CYP1A1 binding site for the APO form (**A**), complex with 3,4,2′-triMS (**B**), complex with 3,4,2′,4′-tetraMS (**C**), and complex with 3,4,2′,4′,6′-pentaMS (**D**). The water occupancies were analyzed at isovalues of 0.5 (blue mesh surface) and 0.7 (solid green surface). SRS1—red, SRS2—orange, SRS3—blue, SRS4—yellow, SRS5—magenta, and SRS6—cyan. Heme with a ligand is represented as a stick model, ligand in black.

The hydration of the region between SRS5, SRS6, the C-terminus of the F helix, and the B’s helix is quite varied. In the APO form, the interior of the FG-loop is rich in water molecules with stable positions. Furthermore, two water molecules are visible near the SRS5 region under the FG-loop. In the complex with 3,4-diMS, a loop (cluster) of water molecules is visible between the SRS5 and SRS6, which joins with a chain of waters in the solvent channel. Water is also present in the area between SRS5, SRS6, and the FG-loop in the complexes with 3,4,2′,4′-tetraMS (a long chain of waters) and 3,4,2′,4′,6′-pentaMS (two water molecules). Figure 2 shows the hydration of the CYP1A1 binding site in the APO and ligand-bound forms. 

The occupancy of water molecules at the active site and near the substrate recognition sites may be related to the opening time of the substrate and solvent channels (Table 6).

For the CYP1A1 APO form, channel 2c is opened for 7%, next channel 2ac for 15% and each of channels 4 and S for 4% of the simulation time. In the complexes, mainly the 2ac channel (except for 3,4,2′,4′,6′-pentaMS) and the solvent channel (S) or its sub-channels S1 and S2 are open. Moreover, in complexes, channels 2b (3,4,2’-triMS), 2c (3,4,2′,4′-tetraMS), 2e (3,4,2’-triMS), 2f (3,4,2’-triMS), 3 (3,4,2′,4′,6′-pentaMS), and 4 (3,4,2’-triMS) are opened (Table 6).

Water molecule clusters are formed where the water occupies a stable position (Figure 2, occupancy = 0.7). In the case of CYP1A1 complexes, these are primarily two sites, the first located in the region adjacent to SRS6, helices I and F (entrance to the S channel), and the second along the 2ac channel, between helix B’ and helix G (Figure S4). Clustered water molecules often form hydrogen bonds with ligand atoms. Ligand–protein interactions mediated by branched chains of water molecules are also observed.

**CYP1A1-APO:** From the region between SRS1 and the I helix, a chain of water molecules extends along the I helix to the interior of the binding site. Water molecules in this chain form hydrogen bonds with the amino acids of helix I: Gly316, Asp320, and Thr321. Two water molecules are above the heme iron atom (Figure 2A).

**CYP1A1-3,4,2′-triMS:** Only the fragment occupying the space between the methoxy substituents at the positions 2′ and 3 of 3,4,2′-triMS remained from the chain of water molecules spanning in the APO form along the helix I (Figure 2B). Water molecules formed hydrogen bonds with the oxygen atom of the 2′ substituent, lasting for 43% of the analyzed simulation time. Participating in this H-bond, the water molecules mediate the binding of the ligand also to Asp313 and, to a much lesser extent, to Ser122 (hydrogen bond bridges). Helix I is hydrated in its middle part less than in the APO form.

Water molecules entering the binding site through channel 2f (space between SRS5, SRS6, and helix F) are hydrogen bonded with the 4-methoxy and 3-methoxy substituents. The hydrogen bond between water and the oxygen atom at the 4 position is maintained for 41% of the analyzed time (Table 5). 

**CYP1A1-3,4,2′,4′-tetraMS:** The water molecules entering the active center from the solvent channel (Figure 2C) formed a stable hydrogen bond with the oxygen atom of the methoxy substituents in the 2’ position (maintained for 49% of the analyzed time). Less stable are H-bonds with substituents in positions 4- and 4′ (Table 5). Interestingly, the water molecule bound to the oxygen of the 2′-methoxy substituent for 27% of the simulation time also forms a bridging hydrogen bond with the residue Gly316 of the protein (Table 5).

**CYP1A1-3,4,2′,4′,6′-pentaMS:** The central part of the I helix was highly hydrated and Gly316 and Asp320 formed hydrogen bonds with water molecules. Figure 2D shows water molecules reaching the binding site through the solvent tunnel. This chain/cluster of water molecules extended up to the substituent at position 3 of 3,4,2′,4′,6′-pentaMS, allowing the formation of a hydrogen bond between water and the 3-methoxy group. To a small extent, the water molecule mediates the interaction of 3-methoxy substituent with the protein through the Asn255 amino acid residue. The water molecules in the binding site also formed hydrogen bonds with the oxygen atoms of the 2′, 4′, and 6’ substituents in the ring closer to the heme for 28%, 18%, and 3% of the analyzed time, respectively (Table 5). In the case of a 2′-methoxy substituent, the water molecule mediates ligand binding to Ser122, Asp313, and Ser120. An analogous hydrogen bond bridge with Ser122 appears in the case of the 4’-methoxy substituent.

#### 2.2.3. Hydration of the CYP1A2 Binding Site

**Figure 3:**
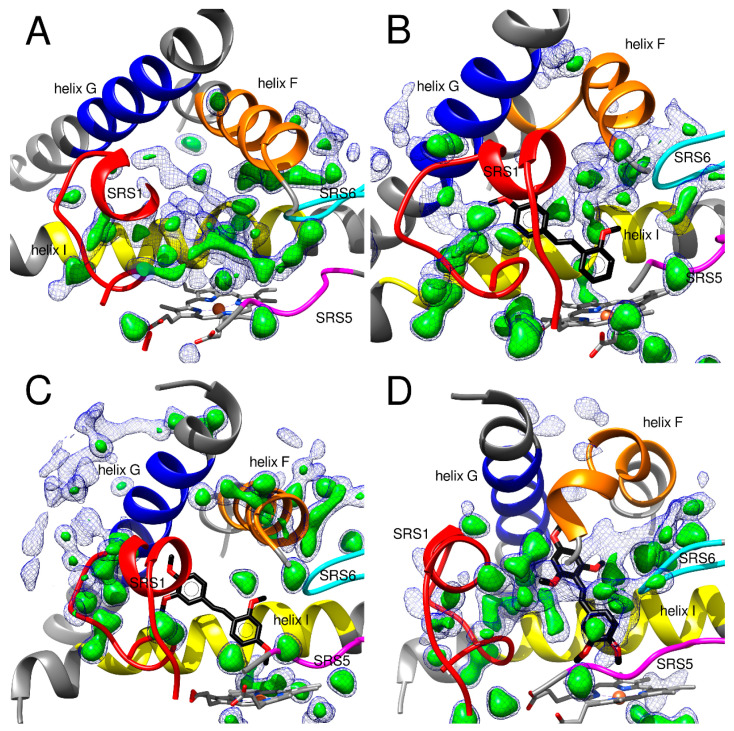
Volumetric maps of water molecule occupancy in the CYP1A2 binding site for the APO form (**A**), complex with 3,4,2′-triMS (**B**), complex with 3,4,2′,4′-tetraMS (**C**), and complex with 3,4,2′,4′,6′-pentaMS (**D**). The water occupancies were analyzed at isovalues of 0.5 (blue mesh surface) and 0.7 (solid green surface). SRS1—red, SRS2—orange, SRS3—blue, SRS4—yellow, SRS5—magenta, and SRS6—cyan. Heme with a ligand is represented as a stick model, ligand in black.

Figure 3 shows the hydration of the CYP1A2 binding site in the APO and ligand-bound forms. In the CYP1A2-APO structure and the structures complexed with the studied methoxystilbenes, particularly in CYP1A2–3,4,2′,4′,6′-pentaMS, many water molecules occupy the region of SRS1 and the space between the BC-loop and the I helix (Figure 3D). The hydrogen bonds are formed with Arg108 (APO and all complexes), Pro109 (APO, 3,4,2′-triMS, 3,4,2′,4′,6′-pentaMS), Asp110 (APO and all complexes), Tyr112 (APO and all complexes), Tyr113 (APO), Ser114 (APO and all complexes), Thr115 (APO and all complexes), Ile117 (3,4,2′-triMS, 3,4,2′,4′-tetraMS), Thr118 (APO, 3,4,2′,4′-tetraMS), Asp119 (APO and all complexes), Gly120 (APO), Gln121 (APO and all complexes), Ser122 (APO, 3,4,2′-triMS, 3,4,2′,4′-tetraMS), Leu123 (APO and all complexes), Thr124 (APO), Phe125 (APO and all complexes), and Ser126 (APO and all complexes).

The region between the F and I helices and SRS6 (solvent channel) is quite strongly hydrated in the CYP1A2 APO structure. Hydrogen bonds are formed with Asn222, Thr223 (the F helix), and the I helix residues Gly316, Ala317, Phe319, Asp320, Thr321, and Leu497, His501 in the SRS6 region. This area is similarly hydrated in complexes with 3,4,2′-triMS and 3,4,2′,4′,6′-pentaMS (Figure 3B,D) and slightly less hydrated in complex with CYP1A2-3,4,2’,4’-tetraMS. The positions of water molecules existing in this region are relatively stable (occupancy = 0.7), which allows the forming of hydrogen bonds with Asn222 (all complexes), Thr223 (3,4,2′-triMS, 3,4,2′,4′,6′-pentaMS), Asp320 (all complexes), Thr323 (3,4,2′-triMS), Thr385 (all complexes), Ile386 (3,4,2′-triMS), Tyr495 (all complexes), Gly496 (3,4,2′,4′,6′-pentaMS), Leu497 (3,4,2′,4′-tetraMS, 3,4,2′,4′,6′-pentaMS), Thr498 (3,4,2′-triMS), and His501 (all complexes). Detailed information on the water–amino acid hydrogen bonds of the CYP1A2 binding site is presented in Tables S7 and S8 (Supplementary Materials).

In APO form, the space between SRS5 (β1-4), the C-end of the F helix, and the B’ helix is occupied by a long and stable (occupancy = 0.7) chain of water molecules. In CYP1A2-3,4,2’-triMS, the chain is divided into two parts, one at the entrance to the 2f channel and the other within the FG loop. The region between SRS5, SRS6, and the C-terminus of the F helix is the most hydrated in complex with 3,4,2′,4′,6′-pentaMS (Figure 2D) and the water molecules reach deep into its binding site (Figure 3D). In turn, in the complex with 3,4,2’,4’-tetraMS, only two water molecules with stable positions are observed in this area (occupancy = 0.7).

As for CYP1A1, the possibility of water molecules reaching the interior of the active site through channels opening in the protein was investigated. In the APO form of CYP1A2, eight tunnels, 2b, 2c, 2ac, 2e, 2f, 3, 4, and S, were identified (Table 6). Channels 2c and S are often open, for 25% and 23% of the simulation time, respectively. The opening time of the other channels did not exceed 8% of the entire duration of the simulation.

Channel 2c was open in all CYP1A2 complexes (17–36% of simulation time). Apart from the APO form, the S channel was also available in CYP1A2–3,4,2′-triMS and CYP1A2–3,4,2′,4′,6′-pentaMS complexes for 60% and 34% of the simulation time, respectively. Of the eight channels opened in the APO form, all were open in complex with 3,4,2’-triMS, and seven in CYP1A2–3,4,2’,4,6’-pentaMS (Table 6).

As in the case of CYP1A1, clusters of water molecules are formed in places with stabilized water positions (Figure 3, occupancy = 0.7). In CYP1A2 complexes, these places are mainly the 2c and 2e channels, the area between the BC-loop and helix I, and the BC-loop itself (Figure S5). The clusters of water molecules formed there can interact with ligands through hydrogen bonds or mediate interactions with amino acids at the binding site.

**CYP1A2-APO:** In the active center, the water molecules positioned along the I helix form hydrogen bonds with the residues Asp313, Gly316, Ala317, Phe319, Asp320, and Thr321. A stable water molecule above the iron ion of heme was found (Figure 3A; occupancy = 0.7).

**CYP1A2-3,4,2′-triMS:** Water molecules entering the binding site between helices F and I and SRS6 (solvent channel) approach the ligand molecule (Figure 3B). The 2′- and 3-methoxy substituents are bound to water for 36% and 21% of the simulation time, respectively. The bridged H-bond with the protein was observed for 2′- (with Gly316) and 3-methoxy (with Asn257) ligand groups. The oxygen atom of the 4 substituent forms the H-bond with water for only 2% of the analyzed simulation time (Table 5). 

**CYP1A2-3,4,2′,4′-tetraMS:** A short chain of water molecules reaches the enzyme’s active center between the BC-loop and helix G (Figure 3C), forming an H-bond with the 3- and 4-methoxy groups for 39% and 26% of the simulation time, respectively. Both groups also participate in the bridging hydrogen bonds linking the ligand to the protein, the 3-methoxy substituent with Asn312 and 4-methoxy with Asn257. In the ring closer to the heme groups at the 2’ and 4’ positions, hydrogen bonds with water are formed much less often, for 3% and 4% of the analyzed simulation period, respectively (Table 5). 

**CYP1A2-3,4,2′,4′,6′-pentaMS:** Water molecules that enter between the BC-loop and the I helix, through channel 2c (Figure 3D) and from the opposite side through the solvent channel, form H-bonds with the oxygen atoms of the 2′- and 6′-methoxy substituents. In the case of the 2′-methoxy group, the hydrogen bonding lasts for 39% of the simulation time (Table 5). The hydrogen bond with the 6′-methoxy group is stable for 23% of the simulation time, and the water molecule involved in this H-bond mediates the ligand–protein interaction with Asp320. The substituents 3- and 4-methoxy interact with water molecules entering the binding site via channel 2f (between the SRS5 and heme). The hydrogen bonds with their oxygen atoms last for 15% and 13% of the analyzed time for the 3-methoxy and 4-methoxy groups, respectively (Table 5).

#### 2.2.4. Hydration of the CYP1B1 Binding Site

**Figure 4:**
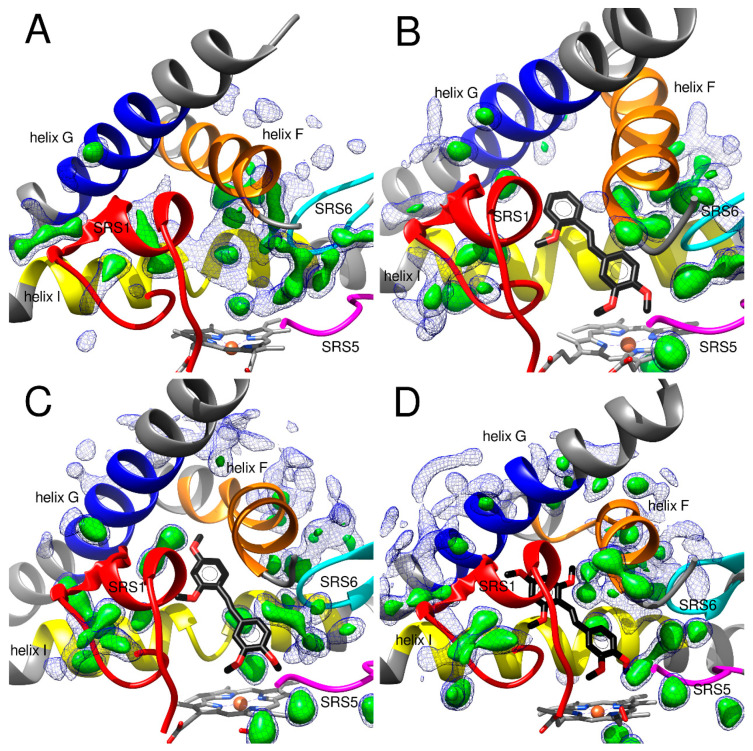
Volumetric maps of water molecule occupancy in the CYP1B1 binding site for the APO form (**A**), complex with 3,4,2′-triMS (**B**), complex with 3,4,2′,4′-tetraMS (**C**), and complex with 3,4,2′,4′,6′-pentaMS (**D**). The water occupancies were analyzed at isovalues of 0.5 (blue mesh surface) and 0.7 (solid green surface). SRS1—red, SRS2—orange, SRS3—blue, SRS4—yellow, SRS5—magenta, and SRS6—cyan. Heme with a ligand is represented as a stick model, ligand in black.

The degree of hydration of the SRS1 region, between the BC-loop and the I and B’ helices, is varied (Figure 4). Interestingly, hydration of this region was the strongest for the complexes, especially for CYP1B1-3,4,2′,4′,6′-pentaMS (Figure 4B–D) and weaker for CYP1B1-APO (Figure 4A). In the CYP1B1-APO structure, water molecules form hydrogen bonds with Ser127, Gly128, Arg130, Ser131 (SRS1), and the two residues of the I helix: Thr325 and Asp326.

In the more hydrated CYP1B1 complexes, water molecules form hydrogen bonds in SRS1 with Ser119 (all complexes), Arg124 (all complexes), Ser127 (3,4,2′-triMS, 3,4,2′,4′-tetraMS), Gly128 (all complexes), Gly129 (3,4,2′-triMS, 3,4,2′,4′-tetraMS), Arg130 (3,4,2′-triMS, 3,4,2′,4′-tetraMS), and Ser131 (all complexes). In the fragment of helix I adjacent to SRS1, Thr325 (3,4,2′,4′,6′-pentaMS), Asp326 (3,4,2′-triMS, 3,4,2′,4′-tetraMS), and Gly329 (3,4,2′,4′-tetraMS, 3,4,2′,4′,6′-pentaMS) are involved in hydrogen bonding with water.

In all CYP1B1 structures, water molecules were found between the B’ helix and the G helix (Figure 4), forming hydrogen bonds with Arg266 or with Asn267 (the G helix) in the APO form and the CYP1B1 complexes.

The region of the solvent tunnel, between the two helices F and I and SRS6, was strongly hydrated in all of the analyzed structures. In CYP1B1’s ligand-free form, the residues Gly329, Gln332, Asp333, and Thr334 of the I helix and His227, Glu229, and Glu230 of the F helix were engaged in hydrogen bonding with water molecules. 

In the tested complexes, hydrogen bonds with water were formed with the I helix residues Ala330 (3,4,2′,4′-tetraMS), Gln332 (3,4,2′-triMS), Asp333 (all complexes), Thr334 (3,4,2′,4′,6′-pentaMS), and Ser336 (3,4,2′-triMS, 3,4,2′,4′-tetraMS). Hydrogen bonds with the residue Thr334 stabilized the water molecule between the I helix and 3-methoxy and 4-methoxy substituents of 3,4,2′,4′-tetraMS. Among the amino acids of helix F, hydrogen bonds with water form with His227 (all complexes), Asn228 (3,4,2’-triMS), Glu229, and Glu230 (all complexes). Detailed information on the water–amino acid hydrogen bonds of the CYP1B1 binding site is summarized in Tables S9 and S10 (Supplementary Materials).

In CYP1B1’s APO form, the areas between the FG-loop and the B’ helix and between the FG-loop, SRS5, and SRS6 were noticeably hydrated. Fewer water molecules than in the APO structure were observed in the CYP1B1 complexed with 3,4,2′-triMS, 3,4,2′,4′-tetraMS and 3,4,2′,4′,6′-pentaMS. In complexes, water molecules were observed only in the area between the FG-loop, SRS5, and SRS6. This area was strongly hydrated by water molecules with stable positions (occupancy = 0.7), both in the APO structure and in the CYP1B1 complexes with 3,4,2′,4′-tetraMS and 3,4,2′,4′,6′-pentaMS water molecules formed hydrogen bonds with Tyr507 and Leu509 of SRS6.

In CYP1B1, as in CYP1A1, many water molecules have a stable position at the entrance to the S tunnel (Figure 4). In turn, opening the 2f channel in complexes results in the appearance of water molecules near the heme-facing ligand ring in the CYP1B1–3,4,2′,4′-tetraMS and CYP1B1–3,4,2′,4′,6′-pentaMS complexes (Figure 4C,D). In both places, that is, at the entrance of the S1/S2 and 2f channels, water molecule clusters or chains appear, interacting with ligands or mediating ligand–protein interactions (Figure S6).

Of note is that the region between the F and G helices, the opening of tunnel 3, is strongly hydrated in the CYP1B1-3,4,2′,4′-tetraMS and CYP1B1-3,4,2′,4′,6′-pentaMS structures (Figure 4C,D). In the CYP1B1 APO, water molecules can enter the active site through channels 2a, 2f, S1/S2, and 3 (Table 6). The complexes are dominated by the 2f channel and the solvent channel (S1 and S2). The solvent channel is branched, and the degree of opening of the individual S1/S2 branches varies depending on the ligand bound (Table 6). 

**CYP1B1-APO:** In the CYP1B1 binding cavity, a chain/cluster of water molecules starting between the B’ and G helices extends along the I helix joining with the cluster of water molecules that enters through the S1 and S2 channels. These waters formed hydrogen bonds with the amino acids of helices I (Asp333, Thr334) and F (His227, Glu229, Glu230). As in the previously discussed APO forms of CYP1A1 and CYP1A2, also in CYP1B1, there is a stable (occupancy = 0.7) water molecule above the heme iron ion (Figure 4A).

**CYP1B1-3,4,2′-triMS:** The unique feature of this complex, among all the CYP1 complexes with trans-stilbene methoxy derivatives analyzed here, is the complete lack of water–ligand hydrogen bonds and the smallest average number of water molecules located at a distance of 3.4 Å and 5.0 Å from the ligand (Table 4). Although water molecules approach the 3,4,2′-triMS molecule from the S and 2c channels (Figure 4B), they do not form hydrogen bonds with this ligand. It should also be noted that 3,4,2′-triMS is a potent and selective inhibitor of CYP1B1 (Table 1).

**CYP1B1-3,4,2′,4′-tetraMS:** A relatively stable hydrogen bond with water was formed with the oxygen atom of the 4-methoxy group (38% of the analyzed simulation time). The water molecule bound to this substituent also mediated the interaction between the ligand and the amino acids Leu509, Ile399, and Thr334 (Table 5). The 4-methoxy group forms a hydrogen bond with the water located in the vicinity of Thr334 (helix I), as well as with water molecules reaching the binding site through/via channel 2f, the area between SRS5, SRS6, and the FG-loop (Figure 4C). The methoxy group in the 3 position was bound to the water molecule only for 1% of the simulation time.

In turn, the hydrogen bond between water and the oxygen atom at the 4′ position lasts the longest, for 49% of the analyzed time (Table 5). Water molecules interacting with the 4’-methoxy group are located between the B’ and G helices (Figure 4C).

**CYP1B1-3,4,2′,4′,6′-pentaMS:** Only three oxygen atoms out of the five in the ligand molecule formed hydrogen bonds with water molecules. The H-bond between the oxygen atom of the 6′-methoxy substituent and a water molecule persisted for 41% of the analyzed time. This water molecule was also involved in a hydrogen bonding bridge with Gln332 or Gly329 (Table 5). The oxygen atom of the 4-methoxy group was engaged in an H-bond with water for 23% of the simulation time. The H-bond with the 3-methoxy group was the least stable (17% of the simulation time). However, both groups participated in bridging hydrogen bonding via water with Ile 399 (Table 5). All these hydrogen bonds may have arisen due to the presence of water molecules in the area between the I and F helices for the 6′-methoxy group and between SRS5, SRS6, and the FG-loop for substituents in the 3 and 4 positions (Figure 4D).

Comparing the hydration of the APO forms and the structures complexed with the ligands with the relevant X-ray protein structures of CYPs complexed with ANF, it should be noted that the positions of water molecules during the analyzed simulation time largely coincide with the areas occupied by water in the crystal structures (Table 7). In the research of Rudling et al. on the impact of ligand binding on hydration networks, the hydration sites identified from MD simulations were reproduced in 73% of the binding water molecules observed in the X-ray structures [39]. In this study, the reproducibility of hydration sites from MD simulations reached 75 to 92 percent.

### 2.3. The High Affinity of 3,4,2′-triMS for the CYP1B1 Active Site

**Figure 5:**
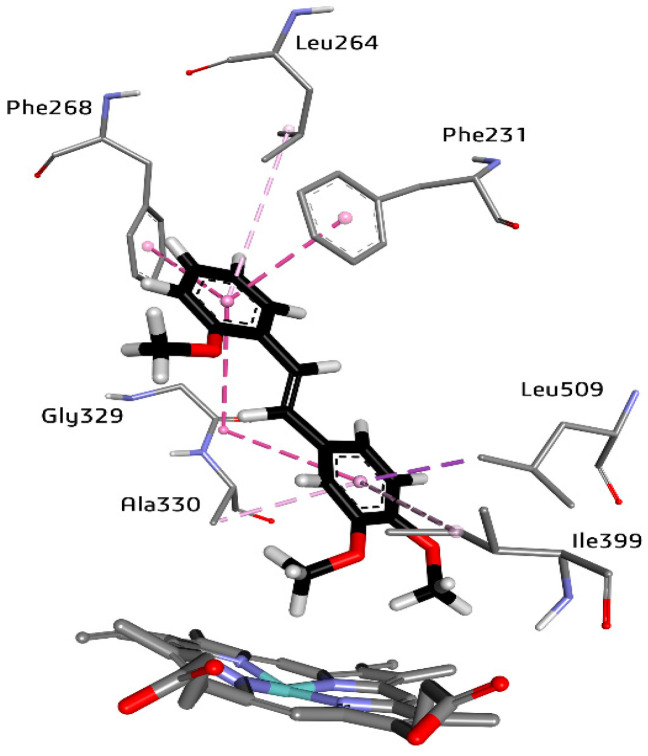
Interactions of 3,4,2′-triMS in the CYP1B1 binding site: π-π with Phe231 and Phe268; π-alkyl with Leu264, Ala330, Ile399, and Leu509; amide-π between the Gly229/Ala330 peptide bond and the ligand aromatic rings. Heme represented as stick model at the bottom.

Our previous studies on the inhibitory activity of methoxy-trans-stilbenes toward CYP1 isozymes revealed an extremely strong affinity of 3,4,2′-triMS toward CYP1B1, an enzyme involved in the metabolism of procarcinogens, which is overexpressed in cancer cells and might be a potential target for cancer therapy. 3,4,2′-triMS inhibited CYP1B1 activity with an IC_50_ value of 4.0 nM, exhibiting 830-fold selectivity for CYP1B1 over CYP1A2 and 90-fold selectivity for CYP1B1 over CYP1A1 (Table 1). Docking studies showed the favorable pose of 3,4,2′-triMS with the A ring directed to the heme and the occurrence of a π-π stacking interaction with Phe231. However, among the other methoxystilbenes studied, 3,4,2′-triMS, characterized by the highest value of binding free energy, docking studies did not indicate other specific interactions of this compound with the CYP1B1 binding site that could explain its high inhibitory activity, despite numerous hydrophobic interactions with amino acid residues of the CYP1B1 binding site [22]. The present studies of ligand–enzyme complexes with the use of MD simulations confirmed the results of the molecular docking studies with respect to hydrophobic interactions. The analysis of the close contacts of ligands in the binding pocket of CYP1B1 in the last ten nanoseconds of the simulation (Supplementary Materials, Table S11) showed more frequent interactions of 3,4,2’-triMS with the hydrophobic side chains of Leu264 and Ala330, than those in CYP1B1 complexes with other ligands. Furthermore, 3,4,2′-triMS was engaged in multiple hydrophobic intermolecular π-π interactions with Phe231 and Phe268, π-alkyl with Leu264, Ala330, Ile399, and Leu509, and amide-π interactions between the Gly229/Ala330 peptide bond and the ligand rings (Figure 5). This special ability of 3,4,2′-triMS to form hydrophobic interactions may result from the lack of hydrogen bonds with water (Table 5) and proteins (Table 2). Moreover, the average number of water molecules in the active site of CYP1B1 complexed with 3,4,2′-triMS within the 3.4 nm distance from a ligand is the lowest among the studied complexes (Table 4). 

The interactions of ligands with amino acid residues in the CYP1B1 active site can change the conformation of the protein, causing the closing of channel 2a, which was open in the APO CYP1B1 form (Table 6). Other substrate channels (2b, 2c, 2ac, and 2e) are also closed, making it difficult for ligands to leave the protein and for entry of potential substrate molecules into the enzyme cavity. In other words, the ligands can block the CYP1B1 active site and inhibit its metabolizing function, most evident in the case of 3,4,2′-triMS. It is worth noting that, at the same time, the solvent channel is open 65% of the time during 200 ns of the simulation (Table 6), allowing water to be pushed out of the enzyme active center.

To summarize, the high 3,4,2′-triMS inhibitory potency seems to be the result of not one but many factors, such as structural fitting to the binding site cavity, hydrophobic interactions with amino acid residues, and conformational changes which help to stabilize the energetically favorable position of 3,4,2′-triMS near the heme and additionally keep the suitable channels open/closed.

## 3. Materials and Methods

### 3.1. Selection of Ligands and Protein Structures

The inhibition of CYP1A1, CYP1A2, and CYP1B1 activities by trans-stilbene derivatives was studied previously with the ethoxyresorufin *O*-deethylase assay [20,21,22]. Molecular docking has been performed for a series of 4′-methylthio-trans-stilbene methoxy derivatives [21], polymethoxy-trans-stilbenes with 3,4-dimethoxy motif [22], and 4′-methoxy-trans-stilbene methylthio derivatives [20].

In this study, four earlier studied trans-stilbene derivatives (Scheme 1) were selected for the MD simulation: 3,4,2′-trimethoxy-trans-stilbene (3,4,2′-triMS), 3,4,2′,4′-tetramethoxy-trans-stilbene (3,4,2′,4′-tetraMS), and 3,4,2′,4′,6′-pentamethoxy-trans-stilbene (3,4,2′,4′,6′-tetraMS).

### 3.2. Molecular Docking

CYP1–ligand complexes were obtained by docking the ligands into the binding sites of CYP1A1 (PDB: 4I8V) [12], CYP1A2 (PDB: 2HI4) [10], and CYP1B1 (PDB: 3PM0) [11] with the use of the CDOCKER procedure [40] implemented in BIOVIA Discovery Studio 2016 [41].

Receptors were prepared for docking by the ‘prepare protein’ procedure, which removed water molecules, added hydrogen atoms, and protonated amino acid residues at the specified pH (pH = 7.4). A short gap in the structure of CYP1B1 between amino acid residues 308-311, located in the protein loop, was filled and modeled with the ‘Prepare protein’ protocol with the ‘Build loops’ option active. The geometry of the gap was predicted and refined with the looper algorithm [42] based on the SEQRES data.

All calculations were performed with the use of the CHARMm forcefield. Partial charges for receptors and ligands were set according to the Momany–Rone (MR) method. The binding sites for CYP1A1, CYP1A2, and CYP1B1 were defined using the cocrystallized ligand (α-naphthoflavone). For each of the ligands, one complex, with the ligand in the pose with the highest CDOCKER energy score, was subjected to molecular dynamics simulations.

The ability of the CDOCKER procedure to reliably determine the poses of ligands in CYP1 binding sites was confirmed in a previous study [20], where the cocrystallized ligand, ANF, was docked into the CYP1A1, CYP1A2, and CYP1B1 binding sites. For each cytochrome, a conformation of ANF with the highest CDOCKER energy score compared with the X-ray pose of ANF gave the lowest value of RMSD for heavy atoms, equal to 0.44, 0.47, and 0.53 Å for CYP1A1, CYP1A2, and CYP1B1, respectively.

### 3.3. Molecular Dynamics Simulations

The VMD program, version 1.9.4a53 [43], with the QwikMD plugin [44] was used to prepare the molecular dynamics simulations. All simulations were carried out with NAMD 2.13 [45] using CHARMM36 [46,47] force field parameters and the TIP3P model for water molecules. The parameters for ligands were derived from the CGenFF force field [48,49,50].

The initial structures were solvated in a water box with a 10 Å distance from any protein residue to the boundary. Then, Na^+^ and Cl^–^ ions were added to the system, making up a salt concentration of 0.15 mol·dm^–3^. All simulations were performed in an NPT ensemble (pressure = 1.01325 bar, temperature = 300 K). A cutoff distance of 12 Å and a switching distance of 10 Å was applied for nonbonded interactions. The particle mesh Ewald (PME) method was employed to evaluate the electrostatics. Bonds to hydrogen atoms were constrained with the SHAKE algorithm allowing for a time step of 2 fs. The molecular system was minimized by 2000 steps. After the minimization, the system was heated from 0 to 300 K for 0.29 ns. Subsequently, the system was equilibrated for 1 ns. During these steps, harmonic restraints were applied to the atoms defined by the selection ‘backbone’. Then, 300 ns of free simulations (production run) were performed to obtain the final structures (400 ns for CYP1A2 apo form). For the entire production run trajectories, the RMSD and RMSF of the protein backbone and ligand were calculated. Protein visualizations and all analyses of the resultant trajectories were performed using VMD. Volumetric maps were visualized with UCSF Chimera, developed by the Resource for Biocomputing, Visualization, and Informatics at the University of California, San Francisco, with support from NIH P41-GM103311 [51].

### 3.4. Tunnel Analysis

Protein tunnels potentially accessible to water molecules were analyzed during the production run from 100 to 300 ns (200–400 ns for CYP1A2 APO). Access channels of CYP1A1, CYP1A2, and CYP1B1 protein molecules were studied and visualized using the CAVER Analyst 2.0 beta software [52,53]. For all systems, snapshots of MD simulation trajectories were extracted at every 50 ps, giving each system 4000 snapshots for analysis. The starting point for tunnel searching was set at the center of the binding site. In the case of CYP1–ligand complexes, the ligand molecule was excluded from residues used in the channels search. Due to the size of water molecules, the probe radius was set to 1.4 Å. Default values were used for other parameters, including a clustering threshold of 3.5 Å. The nomenclature presented by Cojocaru [54] was used for the channels.

### 3.5. Averaging Protein Structures, Structural Changes Induced by Ligands, Hydrogen Bond Analysis, Water Molecule Occupancies, and Radius of Gyration of the Binding Sites

For the last ten nanoseconds of each simulation, when the protein backbone stabilization was achieved, a series of analyses were carried out, which included: (i) averaging the protein structure for the analysis of structural changes in the substrate recognition sites (SRS) concerning the appropriate crystallographic structure; (ii) the formation of hydrogen bonds between the ligand and protein, between water and binding site amino acids, and between water and the ligand; and (iii) creating volumetric maps with the average occupancy of water molecules in the binding cavity.

The averaged protein structures were superimposed on the X-ray structure of the respective cytochrome for visual analysis of the structural changes introduced by the binding ligands. The analyses were focused on structural elements in the vicinity of the binding site, helix B’ or BC-loop (including SRS1) and helices F (including SRS2), G (including SRS3), I (including SRS4), as well as the loops where SRS5 and SRS6 are located. The RMSD of the protein backbone between the superimposed averaged and crystallographic structures was also calculated.

The formation of hydrogen bonds was examined using a hydrogen bond plugin of VMD with a 3.3 Å cutoff distance and a 20° cutoff angle. The water occupancies were calculated using the VMD VolMap plugin to analyze the water molecules’ distribution inside the active site and around the ligands The obtained averaged (over the last ten nanoseconds of simulations) volumetric maps (3D grids) were analyzed at isovalues of 0.5 and 0.7.

For estimating the effect of bound ligands on the change in the active site’s size, the averaged value of the radius of gyration (Rg) for the binding site amino acids was calculated in the last nanosecond of the simulations and compared to the corresponding Rg value for the X-ray structure.

## 4. Conclusions

The molecular interactions of selected polymethoxy-trans-stilbene derivatives with the binding sites of CYP1 isozymes were investigated with computational methods. The substituent pattern of ligands significantly influenced the changes in the enzyme structures, affecting the process of substrate penetration into the enzyme and hydration of the active center. The most pronounced structural changes in the CYP1 binding sites were observed in SRS2 (helix F) and SRS3 (helix G), while SRS5 in the studied isozymes for all ligands tested did not differ from the crystallographic structures.

In CYP1–ligand complexes, the number of water molecules at the binding site depended on the cytochrome and the ligand bound. The presence of water in the binding site resulted in the formation of water–protein, water–ligand, and in some cases, bridging ligand–water–protein hydrogen bonds. Direct hydrogen bonds between the studied trans-stilbene derivatives and the active site residues were rarely formed or were unstable. These studies have shown that water molecules are actively involved in the ligand–enzyme interactions and, in this way, can modulate the affinity of substrates/inhibitors to the active sites of studied cytochromes.

Another factor governing the inhibitor specificity and potency was found to be the channel opening time. The analysis of channel opening during the simulation showed significant differences between the studied cytochromes; the biggest differences were observed for the solvent channel and channels 2c, 2ac, and 2f; however, determining which of the channels plays a special role is difficult due to the complexity of the enzyme structural changes induced by ligand–protein interactions.

The computational methods used in this study have provided better insight into the processes taking place in the active center of cytochromes and, thus, may prove useful in designing effective inhibitors of the enzymes that are targets of chemoprevention and cancer therapy.

## Figures and Tables

**Table 1:** Selective inhibition of CYP1A1, CYP1A2, and CYP1B1 activities by polymethoxy-*trans*-stilbene derivatives. The IC_50_ values were determined with 7-ethoxyresorufin as a substrate (0.5 μM) and published previously [20,22].

Compound	IC_50_ (95% Confidence Interval) (μM)			Ratio	
	CYP1A1	CYP1A2	CYP1B1	CYP1A1/CYP1B1	CYP1A2/CYP1B1
3,4,2′-triMS	0.36 (0.27–0.48)	4.93 (4.43–5.50)	0.0034 (0.0016–0.0072)	90	830
3,4,2′,4′-tetraMS	0.51 (0.24–0.78)	>100	0.21 (0.14–0.28)	2.4	>480
3,4,2′,4′,6′-pentaMS	0.27 (0.18–0.36)	>100	0.30 (0.25–0.35)	0.9	>333
ANF *	0.01	0.0038	0.0013	7.7	2.9

* minimum IC_50_ values for α-naphthoflavone (ANF); the data comes from the BindingDB database.

**Table 2:** Characteristics of hydrogen bonds formed between the oxygen atoms of methoxy-trans-stilbenes and CYP1 isoenzymes: CYP1A1, CYP1A2, and CYP1B1. The occupancy expresses the percent of time when an H-bond was observed within the last ten nanoseconds of the MD simulation.

Ligand	CYP1A1			CYP1A2			CYP1B1		
	Amino Acid	Substituent	Occupancy %	Amino Acid	Substituent	Occupancy %	Amino Acid	Substituent	Occupancy %
3,4,2′-triMS	Ser122	3-methoxy	0.26	Asn257	4-methoxy	10.74	–	–	–
3,4,2′,4′-tetraMS	–	–	–	–	–	–	Ile399	4-methoxy	0.02
3,4,2′,4′,6′-pentaMS	Asn255 Asn255 Ser122	4-methoxy 3-methoxy 2′-methoxy	27.32 0.04 0.02	Thr124	3-methoxy	0.40	Thr334	4-methoxy	0.10

**Table 3:** The radius of gyration (Å) of the binding site amino acids calculated for X-ray and simulated structures (in parentheses: the increase in Rg in percent from the value for the crystal structure).

Structure/Complex	CYP1A1	CYP1A2	CYP1B1
X-ray	8.53	8.41	8.65
APO	8.86 (+4%)	9.12 (+8%)	9.09 (+5%)
3,4,2′-triMS	9.11 (+7%)	9.19 (+9%)	8.93 (+3%)
3,4,2′,4′-tetraMS	8.85 (+4%)	8.91 (+6%)	9.06 (+5%)
3,4,2′,4′,6′-pentaMS	9.10 (+7%)	9.51 (+13%)	9.31 (+8%)

**Table 4:** Hydration of CYP1 binding sites complexed with the ligands. The average number of water molecules within the distances of 3.4 and 5.0 Å from the ligand in CYP1A1, CYP1A2, and CYP1B1 complexes (averaged over the last ten nanoseconds of the simulations).

Ligand	Number of Water Molecules					
	CYP1A1		CYP1A2		CYP1B1	
	3.4 Å	5.0 Å	3.4 Å	5.0 Å	3.4 Å	5.0 Å
3,4,2′-triMS	4.7	8.5	5.1	10.5	3.6	5.7
3,4,2′,4′-tetraMS	4.4	8.5	3.5	5.8	6.5	9.0
3,4,2′,4′,6′-pentaMS	5.3	9.7	8.0	15.9	5.4	10.5

**Table 5:** Characteristics of hydrogen bonds formed between the ligands, water, and CYP1 isoenzymes. The occupancy expresses the percent of the time when the H-bond was observed within the last ten nanoseconds of the MD simulation. Ligand–water H-bonds were denoted as ‘Lig-Wat’ and bridged interactions ligand–water–protein as ‘Lig-Wat-Prot’.

Ligand	Substituent	H-Bond Occupancy (%)					
		CYP1A1		CYP1A2		CYP1B1	
		Lig-Wat	Lig-Wat-Prot	Lig-Wat	Lig-Wat-Prot	Lig-Wat	Lig-Wat-Prot
3,4,2′-triMS	3-methoxy	7		21	8 (Asn257)	0	
	4-methoxy	41		2		0	
	2′-methoxy	43	2 (Ser122) 15 (Asp313)	36	8 (Gly316)	0	
3,4,2′,4′-tetraMS	3-methoxy			39	1 (Asn257) 6 (Asn312)	1	
	4-methoxy	2		26	11 (Asn257)	38	2 (Thr334) 5 (Ile399) 12 (Leu509)
	2′-methoxy	49	27 (Gly316)	3			
	4′-methoxy	4		4		49	
3,4,2′,4′,6′-pentaMS	3-methoxy	11	3 (Asn255)	15	2 (Ile386)	17	7 (Ile399)
	4-methoxy			13		23	1 (Ile399)
	2′-methoxy	28	4 (Ser120) 16 (Ser122) 8 (Asp313)	39			
	4′-methoxy	18	4 (Ser122)				
	6′-methoxy	3		23	6 (Asp320)	41	6 (Gly329) 17 (Gln332)

**Table 6:** Percentage of channels opening between 100 and 300 ns or 200 and 400 ns (1A2 APO) of MD simulations for APO forms and bound states of CYP1A1, CYP1A2, and CYP1B1. For branched solvent channel S, sub-channel S1 opens closer to the I helix, while sub-channel S2 opens closer to channel 2f.

	2a	2b	2c	2ac	2e	2f	3	4	S/S1	S2
**CYP1A1**										
APO			7%	15%				4%	4%	
3,4,2′-triMS		7%		70%	5%	39%		3%	18%	12%
3,4,2′,4′-tetraMS			2%	67%					11%	4%
3,4,2′,4′,6′-pentaMS							2%		51%	
**CYP1A2**										
APO		8%	25%	1%	1%	4%	1%	5%	23%	
3,4,2′-triMS		1%	17%	5%	5%	1%	2%	10%	60%	
3,4,2′,4′-tetraMS			36%		1%	5%				
3,4,2′,4′,6′-pentaMS		3%	30%		3%	9%	2%	1%	34%	
**CYP1B1**										
APO	20%					25%	3%		3%	10%
3,4,2′-triMS			3%			15%	8%		65%	9%
3,4,2′,4′-tetraMS						31%	3%		1%	9%
3,4,2′,4′,6′-pentaMS						27%	1%		49%	27%

**Table 7:** The number of water molecules present in X-ray structures of CYP1s within 12 Å from ANF that overlapped areas with a calculated water occupancy greater than 0.5. The number of water molecules compared to the X-ray structure (in percent) is shown in parentheses.

Structure/Complex	CYP1A1	CYP1A2	CYP1B1
X-ray	10 (100%)	32 (100%)	13 (100%)
APO	8 (80%)	28 (87%)	11 (85%)
3,4,2′-triMS	9 (90%)	25 (78%)	11 (85%)
3,4,2′,4′-tetraMS	8 (80%)	25 (78%)	11 (85%)
3,4,2′,4′,6′-pentaMS	8 (80%)	24 (75%)	12 (92%)

## Data Availability

Not applicable.

## Supplementary Materials

The supporting information can be downloaded at: https://www.mdpi.com/article/10.3390/ijms241411481/s1.
