# Supplementary material for: Hydration and Structural Adaptations of the Human CYP1A1, CYP1A2, and CYP1B1 Active Sites by Molecular Dynamics Simulations
Source: Int J Mol Sci. 2023 Jul 14;24(14):11481. doi: 10.3390/ijms241411481 (PMC10380238; doi:10.3390/ijms241411481)
Supplement: Supplementary file 1 [file ijms-24-11481-s001.zip › ijms-2505994-supplementary.pdf]

Supplementary Materials

Hydration and structural adaptations of the human CYP1A1, CYP1A2 and CYP1B1 active sites by molecular dynamics simulations

Zbigniew Dutkiewicz[1], Renata Mikstacka[2]

[1] Department of Chemical Technology of Drugs, Poznan University of Medical Sciences, Grunwaldzka 6, 60-780 Poznań, Poland, zdutkie@ump.edu.pl

[2] Department of Inorganic and Analytical Chemistry, Nicolaus Copernicus University, Collegium Medicum, Dr A. Jurasza 2, 85-089 Bydgoszcz, Poland, mikstar@cm.umk.pl

Keywords: cytochrome P450, CYP1A1, CYP1A2, CYP1B1, molecular docking, molecular dynamics simulation studies, polymethoxy-*trans*-stilbenes

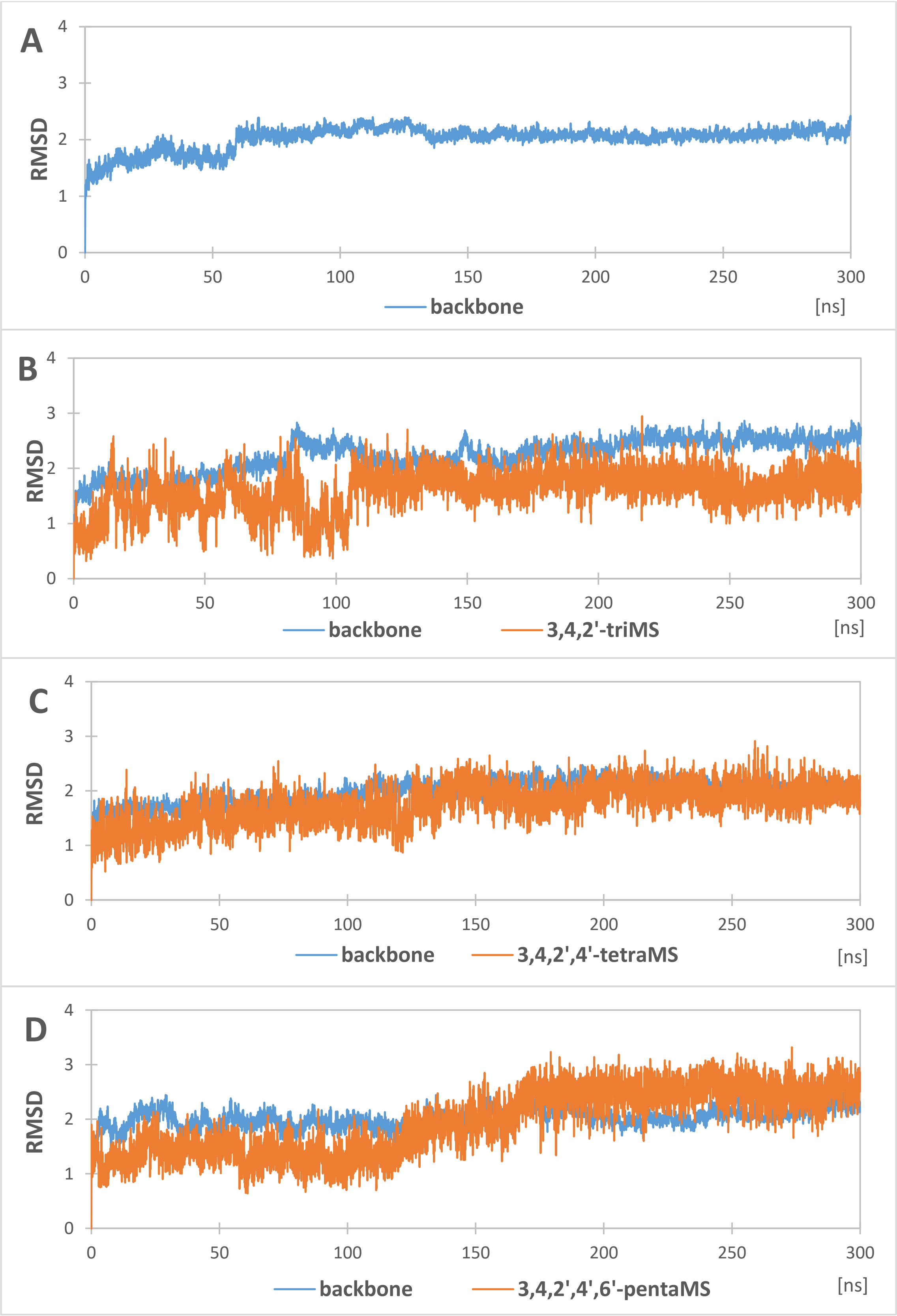

Figure S1. Root mean square deviation of protein backbone (blue line) and ligand (orange line) for CYP1A1 APO (A) and CYP1A1 complexed with 3,4,2’-triMS (B), 3,4,2’,4’-tetraMS (C) and 3,4,2’,4’,6’-pentaMS (D).

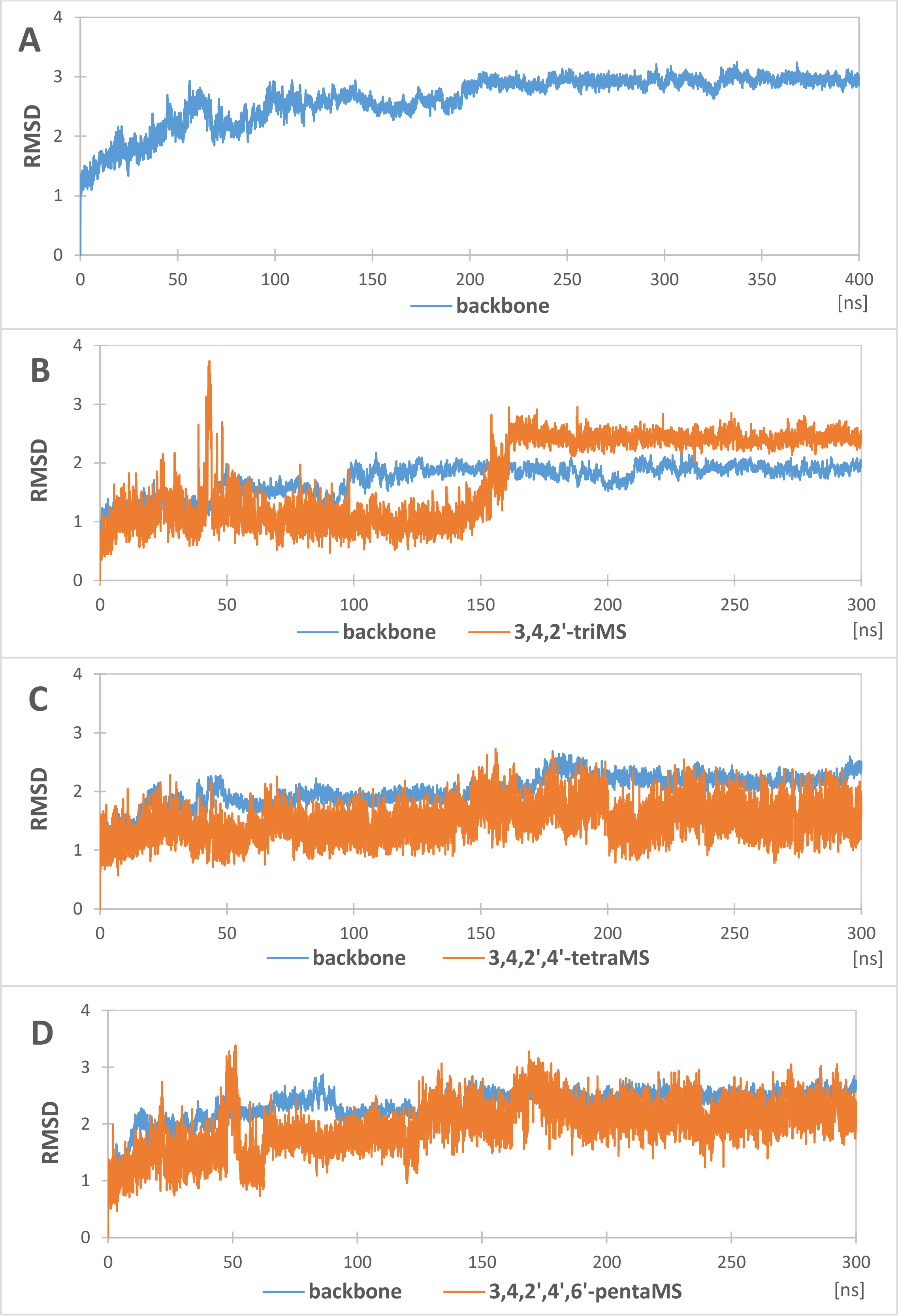

Figure S2. Root mean square deviation of protein backbone (blue line) and ligand (orange line) for CYP1A2 APO (A) and CYP1A2 complexed with 3,4,2’-triMS (B), 3,4,2’,4’-tetraMS (C) and 3,4,2’,4’,6’-pentaMS (D).

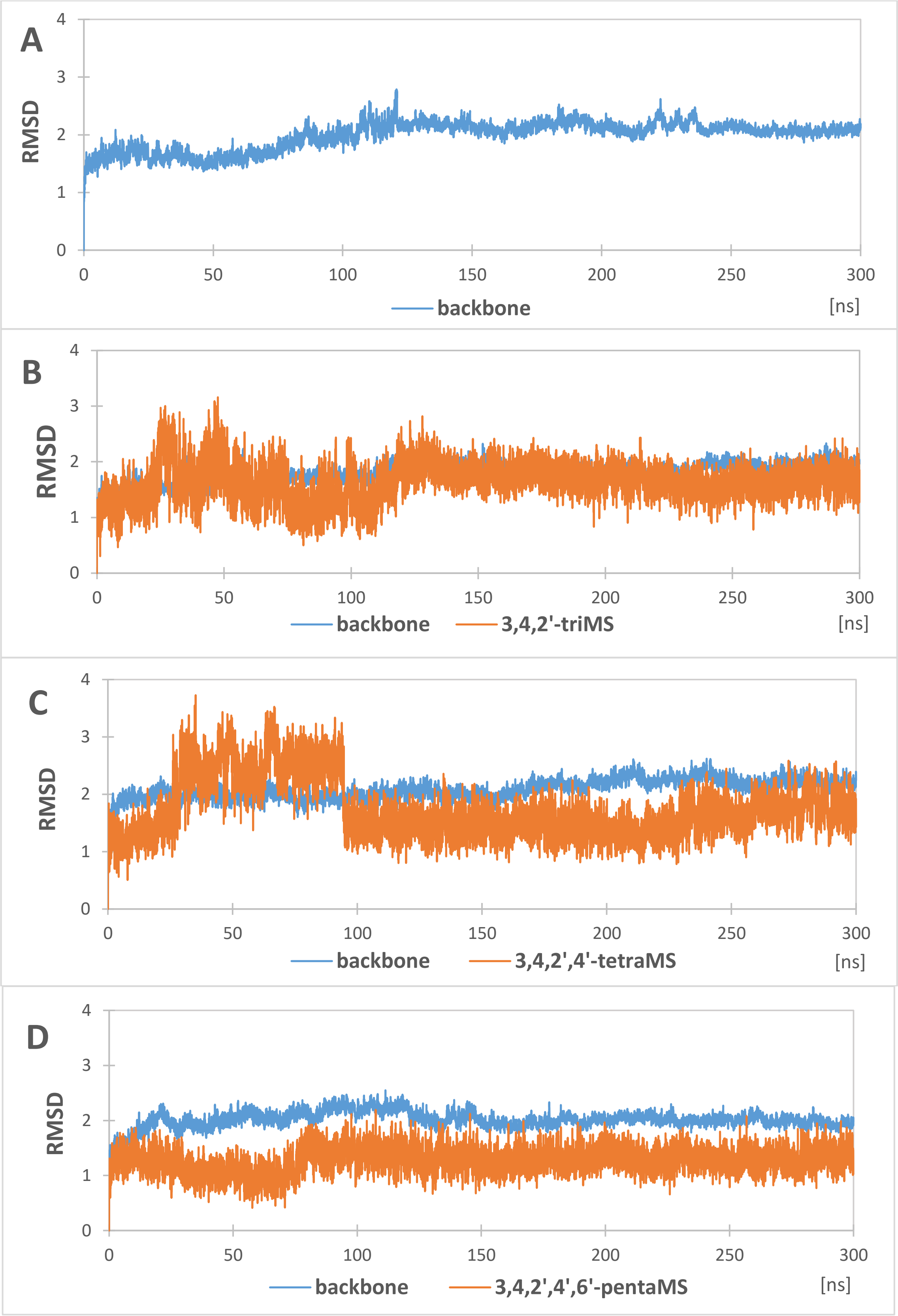

Figure S3. Root mean square deviation of protein backbone (blue line) and ligand (orange line) for CYP1B1 APO (A) and CYP1B1 complexed with 3,4,2’-triMS (B), 3,4,2’,4’-tetraMS (C) and 3,4,2’,4’,6’-pentaMS (D).

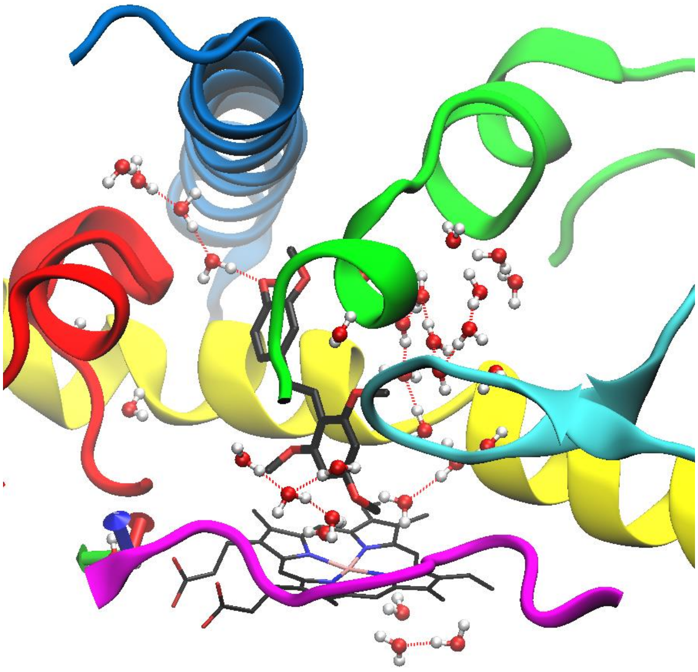

Figure S4. Clusters and chains of water molecules in CYP1A1 complexed with 3,4,2’,4’,6’-pentaMS. Structural elements of CYP1A1: BC-loop and B’ helix – red, helix F – green, helix G – blue, helix I – yellow, SRS5 – magenta, and SRS6 – cyan.

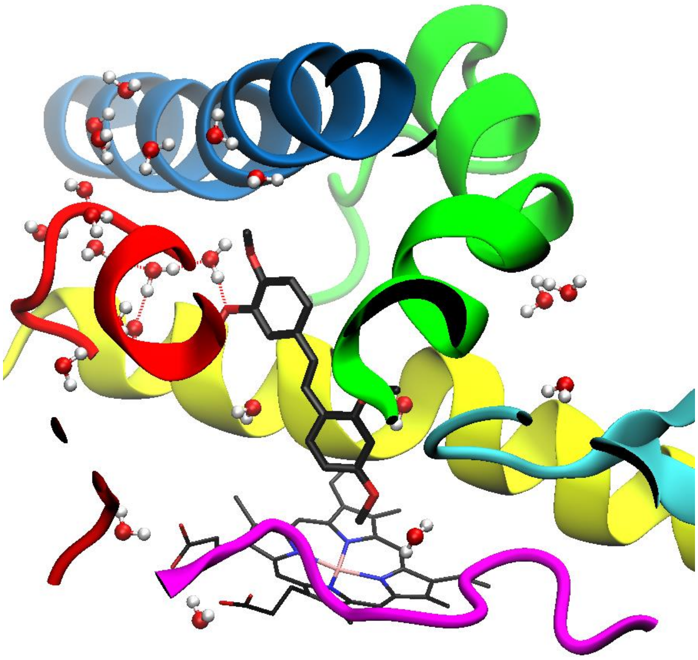

Figure S5. Clusters and chains of water molecules in CYP1A2 complexed with 3,4,2’,4’-tetraMS. Structural elements of CYP1A2: BC-loop and B’ helix – red, helix F – green, helix G – blue, helix I – yellow, SRS5 – magenta, and SRS6 – cyan.

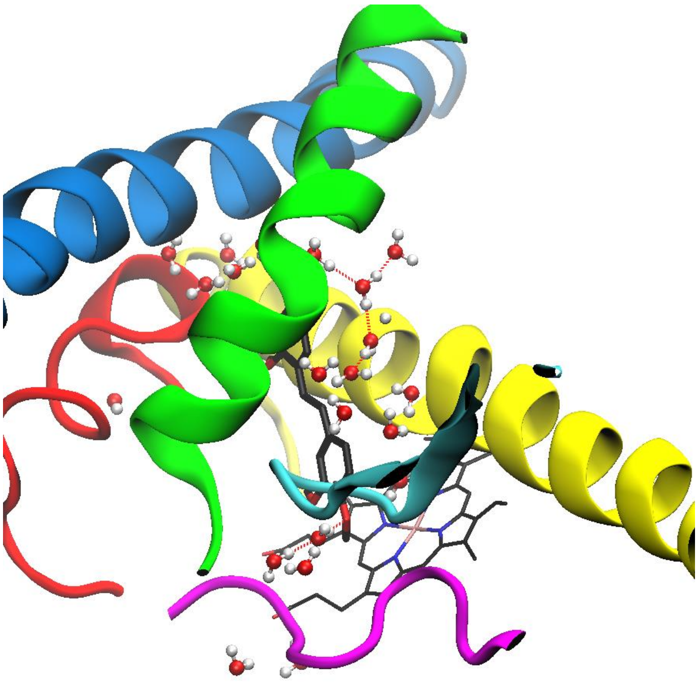

Figure S6. Clusters and chains of water molecules in CYP1B1 complexed with 3,4,2’-triMS. Structural elements of CYP1B1: BC-loop and B’ helix – red, helix F – green, helix G – blue, helix I – yellow, SRS5 – magenta, and SRS6 – cyan.

Table S1. Structural changes in substrate recognition sites of CYP1 isozymes complexed with polymethoxy-*trans*-stilbenes and APO forms.

Region	CYP1A1	CYP1A2	CYP1B1
SRS1	The B′ helix is slightly moved away from helix G in complex with **3,4,2′-triMS** and to a lesser extent in complex with **3,4,2′,4′-tetraMS**.	B′C-loop and helix B′ are slightly moved away from helix G in **all complexes**.	A slight distortion in the helical structure of helix B' in the Arg124-Gly129 range is observed in **all complexes**.
SRS2	Helix F is stretched between Asn219 and Val228 in a structure complexed with **3,4,2′-triMS**.<br>In complex with **3,4,2′,4′-tetraMS,** the region Gln212-Asn221 of F helix has visibly moved away from the I helix.<br>In the case of **3,4,2′,4′,6′-penta MS,** five residue disruption of helix F is shortened to three residues from Asn221 to Asn223.	In a complex with **3,4,2'-triMS**, the distance between the F and I helices increases due to the shift of the F helix (Leu217-Ala230).<br>Minor changes in the helical structure of helix F, in the Ser218-His224 range, are observed in the complex with **3,4,2',4'-tetraMS**.<br>The bound **3,4,2′,4′,6′-pentaMS** ligand causes changes in the F helix between Lys221 and Thr229, increasing the distance between the F helix and SRS6, compared with the X-ray structure.	In all complexes, the ends of helix F are slightly away from helix I than in the X-ray structure. In complexes with **3,4,2'-triMS** and **3,4,2',4',6'-pentaMS**, these are the N-terminal and C-terminal, respectively.<br>In the complex with **3,4,2',4'-tetraMS**, the F helix is bent, and both ends are shifted towards the G helix.
SRS3	In complexes with z **3,4,2′,4′-tetraMS** and **3,4,2′,4′,6′-penta MS**, helix G approaches helix F from Lys257 to Lys264.<br>The N-terminus of helix G is slightly away from the helix F in complexes with **3,4,2′-triMS** and **3,4,2′,4′,6′-penta MS**.	In **all complexes,** the N-terminal part of helix G, between Pro248-Arg259, moves slightly away from helix F towards helix B'.	In complex with **3,4,2'-triMS** helix G, between Asn267 and Asp274, bends towards helix F.<br>The N-terminal helix G is slightly more distant from the F helix than in the X-ray structure in complexes with **3,4,2′,4′-tetraMS** and **3,4,2′,4′,6′-penta MS,** while C-terminal region does not change its position. This shift is most pronounced for the complex with **3,4,2′,4′-tetraMS**.
SRS4	In **all complexes**, slight deformation of helix I in the range Asp304-Leu312 moves it away from SRS1 (B' helix).<br>Additionally, in complex with **3,4,2′-triMS** from Gly316 to Thr324, helix I move towards the binding site.	Within Val311-Asp320, there is a slight deformation of helix I in complexes with **3,4,2'-triMS** and **3,4,2',4'-tetraMS**.	In **all complexes,** there is a slight disturbance of the helical structure of helix I within Gly329-Thr334, most pronounced for the complex with **3,4,2',4'-tetraMS**.

Region	CYP1A1	CYP1A2	CYP1B1
SRS5	In **all complexes,** the SRS5 coincides with the X-ray structure.	In **all complexes,** the SRS5 coincides with the X-ray structure.	In **all complexes**, the SRS5 coincides with the X-ray structure.
SRS6	In complex with **3,4,2'-triMS,** the loop within Pro492-Met498 is moved away from the active center of the enzyme	In a complex with **3,4,2'-triMS,** the loop within Ile494-Met499 has moved away from SRS5 towards helix F.	In complexes with z **3,4,2',4'-tetraMS** and **3,4,2',4',6'-penta MS**, the loop Tyr507-Thr510 approaches the F helix.

Table S2. RMSD values in CYP1A1 SRSs calculated for protein backbone between the averaged structures obtained in the simulations and the reference structure (X-ray). The intensity of the red color in the entire table correlates with the RMSD value.

		APO	3,4,2'-triMS	3,4,2',4'-tetraMS	3,4,2',4',6'-pentaMS
**SRS1**	Arg106	1.326	1.422	0.462	0.547
	Pro107	1.171	1.763	0.402	0.645
	Asp108	1.560	2.905	0.658	0.631
	Leu109	1.161	3.620	0.841	0.657
	Tyr110	1.133	3.906	0.988	0.614
	Thr111	0.568	3.779	0.849	0.257
	Phe112	0.548	3.504	0.971	0.217
	Thr113	0.569	4.671	1.593	0.265
	Leu114	0.526	5.786	1.873	0.491
	Ile115	1.497	4.142	1.092	0.661
	Ser116	2.616	4.116	1.857	1.327
	Asn117	1.893	5.402	3.281	0.827
	Gly118	1.934	4.351	2.929	0.675
	Gln119	2.439	1.728	2.139	1.030
	Ser120	1.955	0.713	1.501	0.920
	Met121	1.800	1.338	1.882	0.424
	Ser122	1.622	1.919	1.639	0.605
	Phe123	0.782	2.044	0.626	0.566
	Ser124	1.342	1.490	1.391	1.083
**SRS2**	Leu217	0.625	2.306	2.930	2.300
	Val218	3.204	2.448	4.837	1.754
	Asn219	3.348	2.339	4.632	2.385
	Leu220	5.561	2.314	2.262	2.972
	Asn221	4.825	2.498	1.591	1.787
	Asn222	2.918	2.939	1.654	2.209
	Asn223	1.550	3.041	1.270	1.954
	Phe224	1.686	3.250	1.322	1.679
	Gly225	1.499	3.893	0.828	2.730
	Glu226	1.209	4.928	0.759	2.964
	Val227	1.284	4.447	0.848	2.112
	Val228	1.046	3.900	0.992	2.432

Table S2. – continued

		APO	3,4,2'-triMS	3,4,2',4'-tetraMS	3,4,2',4',6'-pentaMS
**SRS3**	Phe251	1.239	1.328	0.587	1.503
	Lys252	1.018	1.078	0.849	1.189
	Asp253	1.073	0.669	1.198	2.001
	Leu254	0.948	0.816	1.362	2.091
	Asn255	0.889	1.035	1.474	1.226
	Glu256	1.092	0.952	1.809	0.964
	Lys257	0.824	1.007	2.125	1.683
	Phe258	0.627	1.128	2.170	1.900
	Tyr259	0.878	1.144	2.334	1.351
	Ser260	0.893	1.109	2.581	1.140
	Phe261	0.771	1.225	2.728	1.972
	Met262	0.855	1.050	2.602	2.029
**SRS4**	Asn309	1.905	0.487	0.762	0.625
	Ile310	1.922	0.384	0.975	0.851
	Val311	1.320	0.473	0.471	0.625
	Leu312	1.217	0.804	0.608	1.061
	Asp313	1.411	0.782	0.733	0.965
	Leu314	0.643	0.821	0.452	0.711
	Phe315	0.821	1.030	0.407	0.534
	Gly316	1.716	1.077	0.890	0.636
	Ala317	1.821	1.162	0.896	0.553
	Gly318	1.870	1.438	0.970	0.399
	Phe319	1.876	1.822	1.442	0.195
	Asp320	1.356	1.132	1.007	0.514
	Thr321	1.019	0.584	0.509	0.651
	Val322	0.967	0.563	0.484	0.366
	Thr323	1.042	0.682	0.726	0.407
	Thr324	0.752	0.484	0.617	0.616
**SRS5**	Phe381	1.000	0.578	0.511	0.708
	Val382	1.356	0.752	0.720	0.684
	Pro383	1.418	0.839	0.803	0.617
	Phe384	1.301	0.568	0.905	0.869
	Thr385	1.454	0.880	1.149	1.353
	Ile386	1.238	1.023	0.904	1.163
**SRS6**	Gly495	1.645	4.500	0.906	1.259
	Leu496	1.235	3.445	0.800	0.858
	Thr497	0.702	1.933	0.534	0.784
	Met498	0.580	1.222	0.330	0.662
	Lys499	0.966	1.619	0.177	0.650

Table S3. RMSD values in CYP1A2 SRSs calculated for protein backbone between the averaged structures obtained in the simulations and the reference structure (X-ray). The intensity of the red color in the entire table correlates with the RMSD value.

		APO	3,4,2'-triMS	3,4,2',4'-tetraMS	3,4,2',4',6'-pentaMS
**SRS1**	Arg108	0.746	0.520	0.541	0.469
	Pro109	1.034	0.877	0.692	0.461
	Asp110	1.184	1.235	1.060	0.802
	Leu111	2.151	1.290	1.345	1.471
	Tyr112	2.586	0.863	0.856	1.369
	Thr113	2.161	0.863	1.104	1.349
	Ser114	1.992	1.029	1.428	1.116
	Thr115	2.070	1.182	1.878	1.864
	Leu116	2.055	1.461	2.350	2.904
	Ile117	1.753	1.235	1.605	2.324
	Thr118	1.719	2.562	2.825	2.644
	Asp119	2.179	2.717	2.602	2.193
	Gly120	1.988	2.570	2.947	2.228
	Gln121	1.754	2.030	1.945	1.408
	Ser122	1.501	1.334	1.234	0.923
	Leu123	1.436	1.164	1.087	0.866
	Thr124	1.262	0.992	0.967	0.989
	Phe125	1.712	1.189	1.130	1.318
	Ser126	1.853	1.257	1.021	1.538
**SRS2**	Leu219	2.107	1.918	1.679	3.375
	Val220	3.838	3.259	3.317	5.369
	Lys221	4.725	4.261	4.343	5.089
	Asn222	3.863	4.619	3.477	4.485
	Thr223	2.599	4.696	1.926	5.016
	His224	1.615	4.166	0.839	5.403
	Glu225	3.174	4.211	1.981	6.010
	Phe226	3.535	3.478	1.934	6.081
	Val227	3.002	2.760	1.469	5.284
	Glu228	3.309	2.038	1.573	4.222
	Thr229	4.480	1.775	2.323	3.329
	Ala230	5.040	1.883	2.771	3.329

Table S3. – continued

		APO	3,4,2'-triMS	3,4,2',4'-tetraMS	3,4,2',4',6'-pentaMS
**SRS3**	Phe253	2.963	1.870	1.670	1.374
	Lys254	2.749	1.846	1.571	1.399
	Ala255	2.496	2.377	1.520	2.096
	Phe256	2.190	1.721	1.395	1.656
	Asn257	2.131	0.585	1.262	0.535
	Gln258	2.025	1.153	0.968	0.781
	Arg259	1.640	1.127	0.608	0.916
	Phe260	1.607	0.489	0.365	0.778
	Leu261	1.969	0.555	0.264	0.670
	TRP262	1.706	0.752	0.311	0.507
	Phe263	1.029	0.416	0.453	0.291
	Leu264	1.685	0.343	0.510	0.707
**SRS4**	Asn309	0.944	0.754	0.292	0.647
	Leu310	1.171	0.993	0.718	0.893
	Val311	1.114	0.726	0.879	0.614
	Asn312	0.861	0.657	0.603	0.252
	Asp313	1.572	1.018	1.187	0.844
	Ile314	1.544	0.850	1.378	0.948
	Phe315	1.446	0.873	1.443	0.733
	Gly316	1.896	1.408	1.850	0.752
	Ala317	1.654	1.094	1.821	0.700
	Gly318	1.459	2.188	1.444	0.802
	Phe319	1.965	1.066	1.178	0.631
	Asp320	1.045	1.211	1.008	0.596
	Thr321	0.769	0.900	0.824	0.323
	Val322	0.630	0.697	0.663	0.273
	Thr323	0.337	0.904	0.682	0.213
	Thr324	0.380	0.885	0.719	0.379
**SRS5**	Phe381	0.885	0.925	0.506	0.429
	Leu382	0.877	0.769	0.477	0.568
	Pro383	0.880	0.485	0.498	0.477
	Phe384	0.787	0.868	0.642	0.616
	Thr385	0.812	0.969	0.888	0.653
	Ile386	0.805	0.955	1.112	0.570
**SRS6**	Tyr495	1.990	2.678	1.636	2.093
	Gly496	2.328	2.625	1.417	2.971
	Leu497	2.382	2.232	1.256	2.754
	Thr498	1.764	1.434	0.819	1.469
	Met499	1.065	1.175	0.502	0.852
	Lys500	1.249	1.403	0.986	1.198
	His501	1.138	1.856	1.564	1.427
	Ala502	1.464	2.473	2.157	1.851

Table S4. RMSD values in CYP1B1 SRSs calculated for protein backbone between the averaged structures obtained in the simulations and the reference structure (X-ray). The intensity of the red color in the entire table correlates with the RMSD value.

		APO	3,4,2'-triMS	3,4,2',4'-tetraMS	3,4,2',4',6'-pentaMS
**SRS1**	Arg117	0.419	0.538	0.645	0.424
	Pro118	0.560	0.598	0.652	0.829
	Ser119	0.925	0.856	0.710	1.302
	Phe120	0.990	0.829	0.961	1.446
	Ala121	0.827	0.564	1.173	1.412
	Ser122	0.683	0.411	1.155	1.181
	Phe123	0.868	0.882	1.128	1.225
	Arg124	0.992	1.263	1.381	1.633
	Val125	1.309	1.450	1.420	1.970
	Val126	0.738	0.849	1.515	1.118
	Ser127	0.436	1.153	2.361	0.660
	Gly128	0.403	0.856	2.101	0.790
	Gly129	0.638	0.328	2.387	0.796
	Arg130	0.550	0.611	2.334	0.614
	Ser131	0.693	0.616	1.447	0.605
	Met132	0.882	0.647	1.493	0.661
	Ala133	0.497	0.390	0.755	0.550
	Phe134	0.533	0.334	0.810	0.438
**SRS2**	Leu224	0.562	3.341	1.246	0.835
	Leu225	1.058	3.432	0.580	0.882
	Ser226	1.246	3.354	0.567	1.501
	His227	3.092	2.385	1.111	3.330
	Asn228	3.829	2.009	1.202	3.118
	Glu229	3.695	0.895	1.435	1.886
	Glu230	2.832	0.684	1.801	1.895
	Phe231	2.779	1.250	2.000	2.223
	Gly232	3.024	1.226	2.002	1.729
	Arg233	3.197	0.684	2.050	1.464
	Thr234	3.180	0.390	2.337	1.460
	Val235	2.938	0.866	2.438	1.345

Table S4. – continued

		APO	3,4,2'-triMS	3,4,2',4'-tetraMS	3,4,2',4',6'-pentaMS
**SRS3**	Phe261	2.143	0.344	2.253	0.896
	Glu262	1.928	0.812	1.933	1.026
	Gln263	1.964	1.024	2.363	0.880
	Leu264	1.454	0.549	1.969	0.802
	Asn265	0.876	0.488	1.297	0.821
	Arg266	0.987	0.398	1.344	0.672
	Asn267	0.847	0.513	1.322	0.329
	Phe268	0.665	1.169	0.911	0.596
	Ser269	0.347	1.466	0.531	0.584
	Asn270	0.309	1.452	0.702	0.451
	Phe271	0.572	1.554	0.619	0.424
	Ile272	0.416	1.112	0.293	0.604
**SRS4**	Pro321	0.202	0.713	0.890	0.581
	Ala322	0.127	0.630	0.931	0.462
	Thr323	0.214	0.590	0.831	0.382
	Ile324	0.332	0.620	0.554	0.325
	Thr325	0.555	0.576	0.858	0.335
	Asp326	0.621	0.811	0.986	0.364
	Ile327	0.487	0.669	0.630	0.365
	Phe328	0.938	0.559	1.119	0.956
	Gly329	1.238	0.503	1.487	1.162
	Ala330	1.503	0.778	1.884	1.180
	Ser331	1.124	0.725	1.442	0.926
	Gln332	0.874	0.838	0.996	1.056
	Asp333	1.171	0.508	1.091	1.501
	Thr334	0.866	0.608	0.972	0.987
	Leu335	0.642	0.641	0.821	0.805
	Ser336	0.774	0.756	0.607	1.106
	Thr337	0.712	0.538	0.650	1.010
**SRS5**	Phe394	1.027	0.816	0.917	0.789
	Val395	1.048	0.509	1.059	0.749
	Pro396	0.804	0.360	0.959	0.647
	Val397	0.522	0.583	1.100	0.785
	Thr398	0.366	0.699	0.935	0.922
	Ile399	0.281	0.435	1.023	0.891
**SRS6**	Ser506	1.459	0.796	1.675	2.555
	Tyr507	2.302	1.592	0.782	2.035
	Gly508	2.342	1.422	1.912	2.929
	Leu509	1.970	1.041	1.442	2.704
	Thr510	0.960	1.016	1.125	1.727
	Ile511	0.785	0.670	1.011	1.132
	Lys512	1.430	0.657	1.680	1.866

Table S5. Percentage of the duration (occupancy) of the hydrogen bonding between water molecules and active site amino acids as H-bond acceptors during the last 10 ns of the MD simulation for CYP1A1.

	Acceptor	Occupancy [%]			
		APO	3,4,2'-triMS	3,4,2',4'-tetraMS	3,4,2',4',6'-pentaMS
**SRS1**	Arg106-Main-O	48	39	47	46
	Pro107-Main-O	44	24	33	12
	Asp108-Main-N	< 10	< 10	55	57
	Asp108-Main-O	47	50	91	91
	Asp108-Side-OD1	93	93	93	94
	Asp108-Side-OD2	91	94	< 10	< 10
	Leu109-Main-O	< 10	< 10	< 10	35
	Tyr110-Main-O	< 10	14	< 10	37
	Tyr110-Side-OH	24	34	31	< 10
	Thr111-Side-OG1	23	< 10	29	< 10
	Thr111-Side-OG1	< 10	42	< 10	< 10
	Phe112-Main-O	57	18	22	33
	Thr113-Main-O	42	42	34	47
	Thr113-Side-OG1	60	47	48	51
	Leu114-Main-O	12	44	47	23
	Ile115-Main-O	43	16	54	40
	Ser116-Main-O	< 10	21	17	22
	Ser116-Side-OG	12	< 10	47	< 10
	Asn117-Main-O	36	49	43	40
	Asn117-Side-OD1	53	61	60	31
	Gly118-Main-O	49	11	14	42
	Gln119-Main-O	47	45	27	39
	Gln119-Side-OE1	30	11	52	32
	Ser120-Main-O	35	< 10	< 10	34
	Ser120-Side-OG	< 10	< 10	46	24
	Met121-Main-O	49	50	< 10	< 10
	Ser122-Main-O	< 10	18	< 10	< 10
	Ser122-Side-OG	45	< 10	31	41
	Phe123-Main-O	54	48	39	53
	Ser124-Main-O	< 10	34	< 10	42
	Ser124-Side-OG	36	< 10	51	12

Table S5. - continued

	Acceptor	Occupancy [%]			
		APO	3,4,2'-triMS	3,4,2',4'-tetraMS	3,4,2',4',6'-pentaMS
**SRS2**	Leu217-Main-O	35	11	35	41
	Val218-Main-O	56	56	51	32
	Asn219-Main-O	26	48	49	36
	Asn219-Side-OD1	< 10	54	41	< 10
	Leu220-Main-O	47	44	51	49
	Asn221-Main-O	55	38	42	42
	Asn221-Side-OD1	60	60	52	55
	Asn222-Main-O	16	51	40	55
	Asn222-Side-OD1	51	< 10	16	33
	Asn223-Side-OD1	46	57	60	17
	Phe224-Main-O	16	< 10	< 10	< 10
	Gly225-Main-O	< 10	41	< 10	28
	Glu226-Main-O	48	54	53	55
	Glu226-Side-OE1	90	81	90	94
	Glu226-Side-OE2	91	88	88	94
	Val227-Main-O	75	49	58	49
	Val228-Main-O	37	30	48	< 10
	Val228-Main-O	< 10	< 10	< 10	44
**SRS3**	Lys252-Main-O	27	24	16	< 10
	Asp253-Main-O	< 10	18	27	< 10
	Asp253-Side-OD1	90	91	92	88
	Asp253-Side-OD2	90	93	94	85
	Asn255-Side-OD1	< 10	47	64	22
	Glu256-Main-O	< 10	< 10	12	13
	Glu256-Side-OE1	95	95	95	88
	Glu256-Side-OE2	94	95	95	90
	Lys257-Main-O	12	15	< 10	29
	Tyr259-Side-OH	38	35	34	26
	Ser260-Main-O	< 10	26	< 10	10
	Ser260-Side-OG	44	44	41	40
	Phe261-Main-O	< 10	< 10	< 10	32

Table S5. - continued

	Acceptor	Occupancy [%]			
		APO	3,4,2'-triMS	3,4,2',4'-tetraMS	3,4,2',4',6'-pentaMS
**SRS4**	Asn309-Main-O	< 10	50	53	51
	Asn309-Side-OD1	43	54	< 10	53
	Leu312-Main-O	34	< 10	< 10	< 10
	Asp313-Main-O	30	33	< 10	< 10
	Asp313-Side-OD1	56	81	< 10	60
	Asp313-Side-OD2	70	72	63	61
	Gly316-Main-O	56	< 10	52	51
	Ala317-Main-O	25	< 10	< 10	14
	Phe319-Main-O	12	13	13	< 10
	Asp320-Side-OD1	77	62	72	88
	Asp320-Side-OD2	85	59	84	67
	Thr321-Side-OG1	26	< 10	< 10	< 10
	Thr323-Side-OG1	16	20	11	56
	Thr324-Side-OG1	< 10	< 10	17	< 10
**SRS5**	Phe384-Main-O	< 10	46	18	34
	Thr385-Main-O	52	52	34	< 10
**SRS6**	Gly495-Main-O	< 10	39	27	36
	Thr497-Main-O	15	< 10	< 10	10
	Thr497-Main-O	< 10	< 10	33	28
	Thr497-Side-OG1	< 10	49	15	< 10

Table S6. Percentage of the duration (occupancy) of the hydrogen bonding between water molecules and active site amino acids as H-bond donors during the last 10 ns of the MD simulation for CYP1A1.

		Occupancy [%]			
	**Donor**	**APO**	**3,4,2'-triMS**	**3,4,2',4'-tetraMS**	**3,4,2',4',6'-pentaMS**
**SRS1**	Arg106-Main-N	< 10	26	< 10	< 10
	Arg106-Side-NE	15	61	36	< 10
	Asp108-Main-N	10	47	< 10	45
	Tyr110-Main-N	42	26	29	60
	Tyr110-Side-OH	52	52	52	51
	Thr111-Main-N	33	< 10	43	63
	Thr111-Side-OG1	70	< 10	59	73
	Phe112-Main-N	< 10	23	< 10	< 10
	Thr113-Side-OG1	< 10	< 10	21	22
	Ser116-Main-N	< 10	38	57	< 10
	Ser116-Side-OG	51	< 10	71	73
	Asn117-Main-N	< 10	< 10	20	22
	Asn117-Side-ND2	23	36	40	33
	Gln119-Side-NE2	26	24	27	26
	Ser120-Main-N	< 10	< 10	19	13
	Ser120-Side-OG	63	< 10	< 10	< 10
	Ser122-Main-N	42	16	63	59
	Ser122-Side-OG	51	58	< 10	14
	Ser124-Side-OG	< 10	33	< 10	43
**SRS2**	Asn219-Main-N	50	< 10	< 10	< 10
	Asn219-Side-ND2	39	38	27	47
	Leu220-Main-N	48	21	< 10	< 10
	Asn221-Main-N	< 10	50	43	< 10
	Asn221-Side-ND2	43	38	42	35
	Asn222-Main-N	< 10	42	42	< 10
	Asn222-Side-ND2	27	< 10	63	30
	Asn223-Main-N	< 10	< 10	65	< 10
	Asn223-Side-ND2	25	45	19	39
	Phe224-Main-N	37	< 10	< 10	< 10
	Gly225-Main-N	< 10	36	21	33

Table S6. - continued

	Donor	Occupancy [%]			
		APO	3,4,2'-triMS	3,4,2',4'-tetraMS	3,4,2',4',6'-pentaMS
**SRS3**	Lys252-Side-NZ	80	79	78	71
	Asn255-Side-ND2	21	38	< 10	41
	Lys257-Side-NZ	73	77	33	68
	Tyr259-Side-OH	49	52	51	48
	Ser260-Side-OG	14	49	11	50
**SRS4**	Asn309-Side-ND2	35	30	29	32
	Asp320-Main-N	41	< 10	< 10	< 10
	Thr321-Side-OG1	25	< 10	< 10	< 10
	Thr323-Side-OG1	12	< 10	< 10	< 10
**SRS6**	Gly495-Main-N	< 10	35	< 10	< 10
	Leu496-Main-N	< 10	34	< 10	54
	Thr497-Main-N	43	11	< 10	26
	Thr497-Side-OG1	35	< 10	< 10	29
	Lys499-Main-N	< 10	< 10	19	< 10
	Lys499-Side-NZ	38	41	53	46

Table S7. Percentage of the duration (occupancy) of the hydrogen bonding between water molecules and active site amino acids as H-bond acceptors during the last 10 ns of the MD simulation for CYP1A2.

		Occupancy [%]			
	**Acceptor**	**APO**	**3,4,2'-triMS**	**3,4,2',4'-tetraMS**	**3,4,2',4',6'-pentaMS**
**SRS1**	Arg108-Main-O	44	44	39	41
	Pro109-Main-O	51	57	25	53
	Asp110-Main-O	61	44	52	45
	Asp110-Side-OD1	94	86	88	88
	Asp110-Side-OD2	94	83	85	89
	Leu111-Main-O	31	< 10	< 10	< 10
	Tyr112-Side-OH	39	33	< 10	25
	Thr113-Side-OG1	< 10	31	< 10	< 10
	Ser114-Main-O	36	35	38	29
	Ser114-Side-OG	49	32	19	53
	Thr115-Main-O	49	45	51	45
	Thr115-Side-OG1	47	55	47	48
	Leu116-Main-O	43	< 10	38	< 10
	Ile117-Main-O	22	42	40	29
	Thr118-Main-O	53	23	43	32
	Thr118-Side-OG1	35	< 10	< 10	< 10
	Asp119-Main-O	54	57	51	< 10
	Asp119-Side-OD1	87	81	94	32
	Asp119-Side-OD2	85	89	94	27
	Gly120-Main-O	53	32	34	20
	Gln121-Main-O	21	44	38	46
	Gln121-Side-OE1	54	< 10	11	< 10
	Ser122-Main-O	40	40	42	19
	Leu123-Main-O	60	64	49	55
	Thr124-Side-OG1	50	36	22	38
	Phe125-Main-O	50	53	41	50
	Ser126-Main-O	47	55	54	52
	Ser126-Side-OG	40	23	21	< 10

Table S7. - continued

	Acceptor	Occupancy [%] APO	3,4,2'-triMS	3,4,2',4'-tetraMS	3,4,2',4',6'-pentaMS
**SRS2**	Leu219-Main-O	< 10	41	22	< 10
	Val220-Main-O	< 10	53	< 10	32
	Lys221-Main-O	24	49	< 10	35
	Asn222-Main-O	21	48	13	< 10
	Asn222-Side-OD1	59	61	21	51
	Thr223-Side-OG1	48	37	< 10	51
	His224-Main-O	21	17	25	48
	His224-Side-NE2	45	67	44	70
	Glu225-Main-O	< 10	< 10	< 10	52
	Glu225-Side-OE1	88	88	87	80
	Glu225-Side-OE2	87	87	85	81
	Glu228-Main-O	28	11	36	46
	Glu228-Side-OE1	94	93	94	91
	Glu228-Side-OE2	94	92	93	91
	Thr229-Main-O	48	43	43	28
	Thr229-Side-OG1	50	46	48	44
	Ala230-Main-O	< 10	49	55	55
**SRS3**	Lys254-Main-O	< 10	< 10	16	< 10
	Ala255-Main-O	26	20	22	23
	Asn257-Side-OD1	46	29	23	38
	Gln258-Main-O	26	< 10	20	< 10
	Gln258-Side-OE1	62	70	58	26
	Arg259-Main-O	< 10	33	28	19
	Phe260-Main-O	14	< 10	< 10	< 10
	Trp262-Main-O	10	< 10	< 10	< 10
	Phe263-Main-O	< 10	< 10	22	< 10

Table S7. - continued

	Acceptor	Occupancy [%]			
		APO	3,4,2'-triMS	3,4,2',4'-tetraMS	3,4,2',4',6'-pentaMS
**SRS4**	Asn309-Main-O	16	56	53	43
	Asn309-Side-OD1	62	21	59	28
	Asn312-Main-O	23	< 10	< 10	32
	Asn312-Side-OD1	42	< 10	22	< 10
	Asp313-Main-O	45	< 10	16	22
	Asp313-Side-OD1	66	81	16	78
	Asp313-Side-OD2	58	36	69	63
	Phe315-Main-O	< 10	27	< 10	< 10
	Gly316-Main-O	51	43	38	< 10
	Ala317-Main-O	44	16	31	< 10
	Gly318-Main-O	31	36	< 10	< 10
	Phe319-Main-O	50	< 10	< 10	< 10
	Asp320-Main-O	< 10	< 10	51	82
	Asp320-Side-OD1	81	87	48	62
	Asp320-Side-OD2	80	86	< 10	< 10
	Thr323-Side-OG1	36	56	< 10	19
**SRS5**	Phe384-Main-O	23	30	34	39
	Thr385-Main-O	44	26	54	45
	Thr385-Side-OG1	43	46	41	45
**SRS6**	Tyr495-Main-O	57	61	46	58
	Tyr495-Side-OH	< 10	27	31	11
	Gly496-Main-O	23	14	30	57
	Leu497-Main-N	< 10	32	43	52
	Leu497-Main-O	40	< 10	< 10	< 10
	Thr498-Main-O	33	26	19	< 10
	Thr498-Side-OG1	29	< 10	< 10	32
	His501-Main-O	47	59	56	60
	His501-Side-NE2	36	46	53	49

Table S8. Percentage of the duration (occupancy) of the hydrogen bonding between water molecules and active site amino acids as H-bond donors during the last 10 ns of the MD simulation for CYP1A2.

	**Donor**	**Occupancy [%]**			
		**APO**	**3,4,2'-triMS**	**3,4,2',4'-tetraMS**	**3,4,2',4',6'-pentaMS**
**SRS1**	Arg108-Main-N	< 10	< 10	< 10	21
	Arg108-Side-NH1	< 10	54	< 10	20
	Asp110-Main-N	41	27	25	14
	Leu111-Main-N	10	< 10	< 10	< 10
	Tyr112-Main-N	< 10	45	43	41
	Tyr112-Side-OH	53	51	53	29
	Thr113-Side-OG1	63	< 10	< 10	< 10
	Ser114-Main-N	< 10	< 10	11	23
	Ser114-Side-OG	62	69	48	38
	Thr115-Side-OG1	35	< 10	13	< 10
	Thr118-Main-N	19	< 10	< 10	< 10
	Thr118-Side-OG1	55	< 10	< 10	< 10
	Asp119-Main-N	< 10	< 10	57	36
	Gln121-Side-NE2	30	24	31	< 10
	Ser122-Main-N	23	< 10	< 10	< 10
	Ser122-Side-OG	< 10	< 10	< 10	27
	Thr124-Side-OG1	17	< 10	< 10	< 10
	Ser126-Side-OG	21	34	< 10	25
**SRS2**	Lys221-Side-NZ	80	81	79	82
	Asn222-Main-N	< 10	40	< 10	< 10
	Asn222-Side-ND2	28	36	29	25
	Thr223-Main-N	< 10	30	< 10	< 10
	Thr223-Side-OG1	< 10	49	< 10	< 10
	His224-Main-N	< 10	< 10	< 10	12
	His224-Side-ND1	< 10	26	13	18
	Glu225-Main-N	< 10	44	< 10	< 10
	Phe226-Main-N	< 10	17	< 10	41
	Thr229-Main-N	< 10	< 10	< 10	17
	Thr229-Side-OG1	< 10	10	20	57
**SRS3**	Lys254-Side-NZ	80	62	73	65
	Asn257-Side-ND2	< 10	30	31	17
	Gln258-Side-NE2	33	41	47	< 10
	Arg259-Side-NE	30	36	14	< 10
	Arg259-Side-NH1	68	63	12	43
	Arg259-Side-NH2	34	52	20	22
	Trp262-Side-NE1	32	30	29	< 10

Table S8. - continued

	Donor	Occupancy [%]			
		APO	3,4,2'-triMS	3,4,2',4'-tetraMS	3,4,2',4',6'-pentaMS
**SRS4**	Asn309-Side-ND2	25	30	30	< 10
	Asn312-Side-ND2	48	< 10	46	27
	Phe319-Main-N	< 10	58	< 10	< 10
	Asp320-Main-N	45	< 10	< 10	< 10
	Thr321-Main-N	26	15	< 10	< 10
	Thr323-Side-OG1	58	< 10	< 10	< 10
**SRS5**	ILE386-Main-N	< 10	33	19	16
**SRS6**	Tyr495-Main-N	48	56	39	48
	Tyr495-Side-OH	< 10	32	47	55
	Gly496-Main-N	< 10	< 10	22	10
	Thr498-Main-N	< 10	40	< 10	10
	Thr498-Side-OG1	14	31	< 10	< 10
	Lys500-Main-N	< 10	20	32	36
	Lys500-Side-NZ	16	29	48	42
	His501-Main-N	24	35	25	27
	His501-Side-ND1	< 10	< 10	20	< 10
	Ala502-Main-N	61	11	18	67

Table S9. Percentage of the duration (occupancy) of the hydrogen bonding between water molecules and active site amino acids as H-bond acceptors during the last 10 ns of the MD simulation for CYP1B1.

	Acceptor	Occupancy [%]			
		APO	3,4,2'-triMS	3,4,2',4'-tetraMS	3,4,2',4',6'-pentaMS
SRS1	Arg117-Main-O	20	48	50	54
	Pro118-Main-O	27	22	32	20
	Ser119-Main-O	30	47	45	46
	Ser119-Side-OG	14	44	36	44
	Ala121-Main-O	27	18	< 10	25
	Ser122-Side-OG	40	44	< 10	44
	Phe123-Main-O	38	40	38	29
	Arg124-Main-O	44	46	46	47
	Val125-Main-O	22	14	44	23
	Val126-Main-O	23	6	24	10
	Ser127-Side-OG	39	58	51	27
	Ser127-Main-O	18	39	43	< 10
	Gly128-Main-O	49	40	52	52
	Gly129-Main-O	37	47	49	38
	Arg130-Main-O	23	63	61	< 10
	Ser131-Main-O	61	49	44	72
	Phe134-Main-O	44	50	37	43
SRS2	Leu224-Main-O	11	< 10	< 10	17
	Leu225-Main-O	31	30	45	59
	Ser226-Side-OG	42	47	53	39
	Ser226-Main-O	25	26	45	3
	His227-Side-NE2	69	69	69	73
	His227-Main-N	< 10	< 10	< 10	41
	Asn228-Side-OD1	23	40	< 10	< 10
	Asn228-Main-O	22	30	50	36
	Glu229-Side-OE2	85	89	90	85
	Glu229-Side-OE1	88	91	87	86
	Glu229-Main-O	47	19	11	21
	Glu230-Side-OE2	83	79	87	88
	Glu230-Side-OE1	86	78	85	86
	Gly232-Main-O	< 10	< 10	< 10	13
	Arg233-Main-O	38	41	50	33
	Thr234-Main-O	39	43	< 10	47
	Thr234-Side-OG1	44	< 10	22	< 10
	Val235-Main-O	55	10	44	20

Table S9. - continued

	Acceptor	Occupancy [%]			
		APO	3,4,2'-triMS	3,4,2',4'-tetraMS	3,4,2',4',6'-pentaMS
**SRS3**	Glu262-Side-OE1	87	82	79	88
	Glu262-Side-OE2	88	87	79	80
	Glu262-Main-O	30	< 10	< 10	14
	Gln263-Side-OE1	55	54	29	48
	Leu264-Main-O	16	< 10	< 10	< 10
	Asn265-Side-OD1	42	37	46	17
	Asn265-Main-O	< 10	44	< 10	< 10
	Arg266-Main-O	< 10	27	< 10	< 10
	Asn267-Side-OD1	57	58	60	59
	Asn267-Main-O	35	39	26	28
	Phe268-Main-O	< 10	13	< 10	< 10
	Ser269-Side-OG	48	47	39	45
	Ser269-Main-O	< 10	< 10	31	13
	Asn270-Side-OD1	38	58	65	60
	Asn270-Main-O	19	24	24	23
	Asn270-Main-N	< 10	< 10	< 10	12
**SRS4**	Ala322-Main-O	< 10	51	23	< 10
	Thr325-Side-OG1	53	23	35	53
	Asp326-Side-OD1	55	74	69	< 10
	Asp326-Side-OD2	17	47	70	< 10
	Gly329-Main-O	51	< 10	43	48
	Ala330-Main-O	33	< 10	50	< 10
	Gln332-Side-OE1	53	49	< 10	43
	Asp333-Side-OD2	83	77	85	86
	Asp333-Side-OD1	83	77	79	89
	Thr334-Side-OG1	46	< 10	25	43
	Ser336-Side-OG	2	51	59	< 10
	Thr337-Side-OG1	15	< 10	< 10	41
**SRS5**	Val395-Main-O	< 10	< 10	36	43
	Val397-Main-O	27	14	37	40
	Thr398-Main-O	41	45	49	50
	Thr398-Side-OG1	31	32	31	37
	Ile399-Main-O	< 10	< 10	47	< 10
**SRS6**	Ser506-Side-OG	28	35	37	45
	Tyr507-Main-O	48	23	58	52
	Tyr507-Side-OH	< 10	31	21	46
	Gly508-Main-O	26	38	44	43
	Leu509-Main-O	37	< 10	46	47
	Thr510-Main-O	< 10	41	< 10	< 10
	Thr510-Side-OG1	< 10	< 10	< 10	29

Table S10. Percentage of the duration (occupancy) of the hydrogen bonding between water molecules and active site amino acids as H-bond donors during the last 10 ns of the MD simulation for CYP1B1.

	**Donor**	**Occupancy [%]**			
		**APO**	**3,4,2'-triMS**	**3,4,2',4'-tetraMS**	**3,4,2',4',6'-pentaMS**
**SRS1**	Arg117-Side-NH1	< 10	12	< 10	< 10
	Ser119-Main-N	12	50	47	50
	Ser119-Side-OG	12	46	35	46
	Ala121-Main-N	30	23	< 10	18
	Arg124-Side-NE	14	34	< 10	< 10
	Arg124-Side-NH1	49	59	58	61
	Arg124-Side-NH2	22	60	34	38
	Ser127-Side-OG	< 10	54	71	< 10
	Gly128-Main-N	< 10	< 10	< 10	19
	Arg130-Side-NE	12	18	56	27
	Arg130-Side-NH1	47	48	61	30
	Arg130-Side-NH2	41	42	54	50
	Ser131-Main-N	< 10	< 10	12	< 10
**SRS2**	Ser226-Side-OG	34	39	< 10	47
	His227-Main-N	< 10	< 10	< 10	35
	His227-Side-ND1	19	< 10	< 10	18
	Asn228-Side-ND2	40	28	< 10	59
	Asn228-Side-ND2	< 10	< 10	17	43
	Glu229-Main-N	< 10	< 10	48	< 10
	Arg233-Side-NE	< 10	< 10	< 10	29
	Arg233-Side-NH1	45	25	60	57
	Arg233-Side-NH2	36	36	29	45
	Thr234-Side-OG1	13	< 10	< 10	< 10
**SRS3**	Gln263-Side-NE2	35	37	43	35
	Asn265-Side-ND2	40	38	< 10	29
	Arg266-Side-NE	23	38	38	45
	Arg266-Side-NH1	59	58	63	58
	Arg266-Side-NH2	50	47	46	48
	Asn267-Side-ND2	33	32	42	43
	Ser269-Main-N	< 10	15	< 10	< 10
	Ser269-Side-OG	< 10	27	46	< 10
	Asn270-Side-ND2	48	40	50	45

Table S10. -continued

	Donor	Occupancy [%]			
		APO	3,4,2'-triMS	3,4,2',4'-tetraMS	3,4,2',4',6'-pentaMS
**SRS4**	Thr323-Side-OG1	< 10	74	< 10	42
	Asp333-Main-N	< 10	< 10	11	< 10
	Thr334-Main-N	22	< 10	40	19
	Thr334-Side-OG1	32	< 10	53	< 10
	Ser336-Side-OG	42	< 10	< 10	36
	Thr337-Side-OG1	< 10	< 10	< 10	15
**SRS6**	Ser506-Main-N	< 10	< 10	12	19
	Ser506-Side-OG	39	36	< 10	< 10
	Tyr507-Main-N	38	43	29	43
	Tyr507-Side-OH	42	48	46	41
	Gly508-Main-N	< 10	28	58	75
	Leu509-Main-N	20	< 10	49	38
	Thr510-Main-N	20	38	11	22
	Thr510-Side-OG1	43	35	38	45
	Lys512-Side-NZ	33	18	34	49

Table S11. Percentage of the duration (occupancy) of close contacts between ligands and active site amino acids during the last 10 ns of the MD simulation for CYP1B1.

	Occupancy [%]		
**Residue**	**3,4,2'-triMS**	**3,4,2',4'-tetraMS**	**3,4,2',4',6'-pentaMS**
Arg-117	0.0	0.0	6.6
Val-126	39.7	40.2	84.5
Ser-127	13.8	48.8	93.7
Ser-131	61.3	0.0	34.5
Ala-133	19.1	1.8	22.8
Phe-134	95.6	73.0	96.9
Leu-224	0.0	89.0	0.0
Leu-225	0.0	0.5	0.0
His-227	0.0	21.7	0.0
Asn-228	47.9	96.5	19.6
Phe-231	91.0	99.8	99.9
Leu-264	95.1	11.0	51.1
Asn-265	0.0	0.0	99.8
Phe-268	98.9	89.8	99.9
Ser-269	0.0	0.0	65.0
Thr-325	46.0	0.0	80.9
Asp-326	98.8	0.0	99.9
Gly-329	98.7	100.0	100.0
Ala-330	99.4	64.7	82.6
Gln-332	8.8	48.3	1.6
Asp-333	47.5	41.9	0.0
Thr-334	97.3	94.7	84.6
Val-395	54.3	47.8	13.7
Val-397	0.0	0.2	0.0
Thr-398	0.0	0.3	0.0
ILE-399	98.4	97.7	85.2
Leu-509	90.8	95.3	83.9
Thr-510	34.6	19.9	4.5
HEME	99.9	92.6	97.6

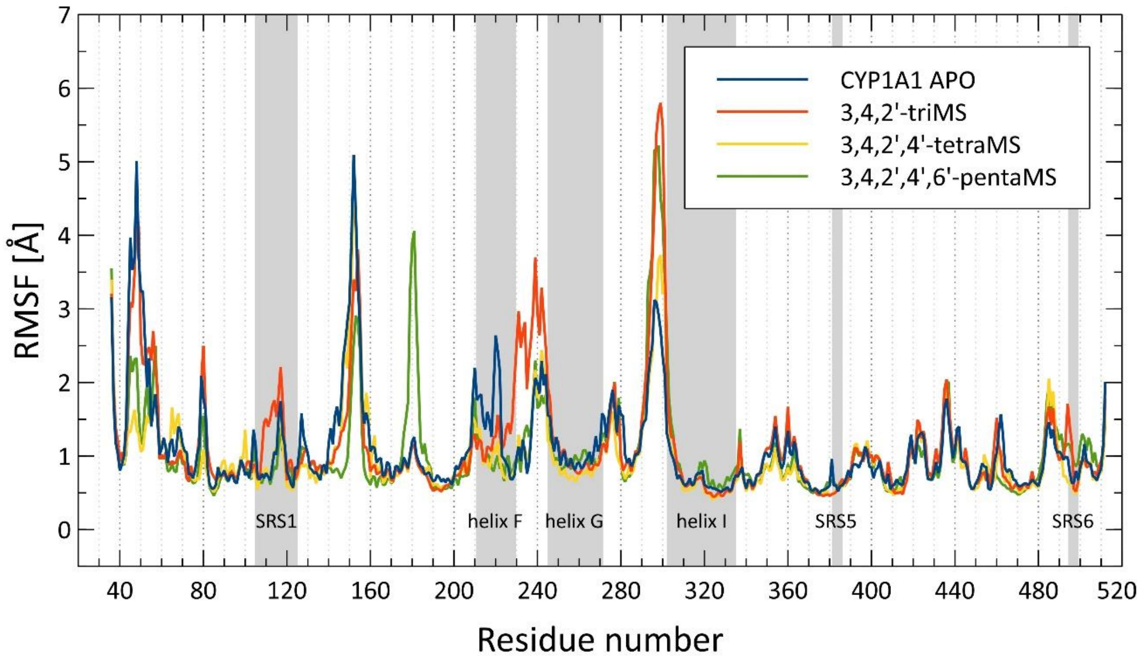

Figure S7. Root mean square fluctuations (RMSF) of protein backbone during MD simulations for CYP1A1 structures.

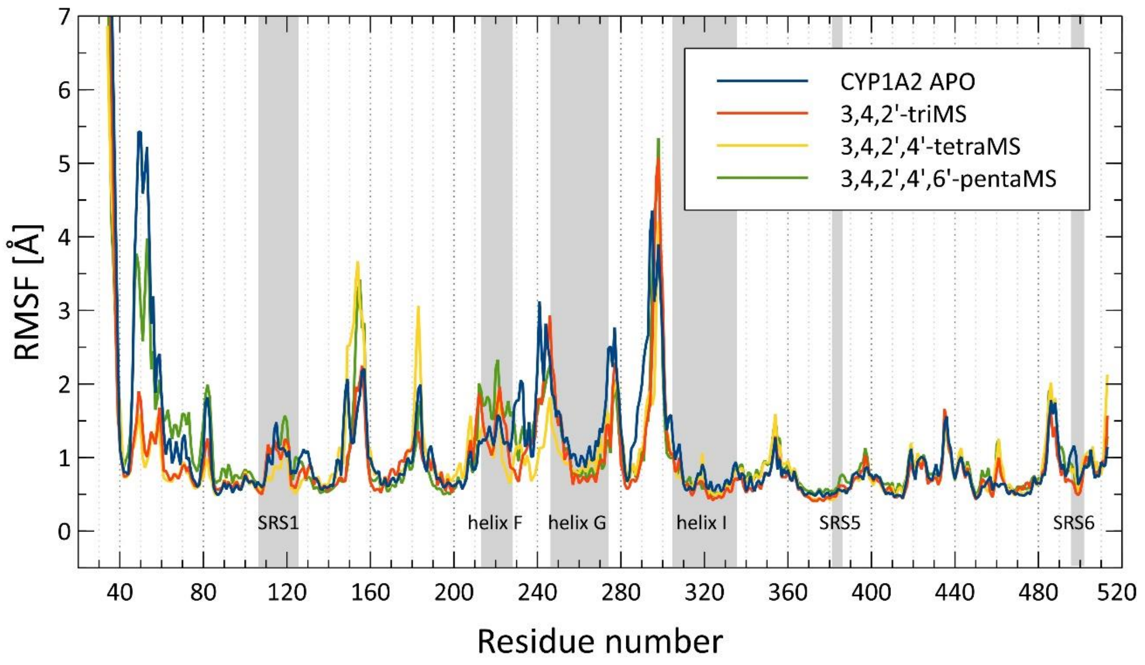

Figure S8. Root mean square fluctuations (RMSF) of protein backbone during MD simulations for CYP1A2 structures.

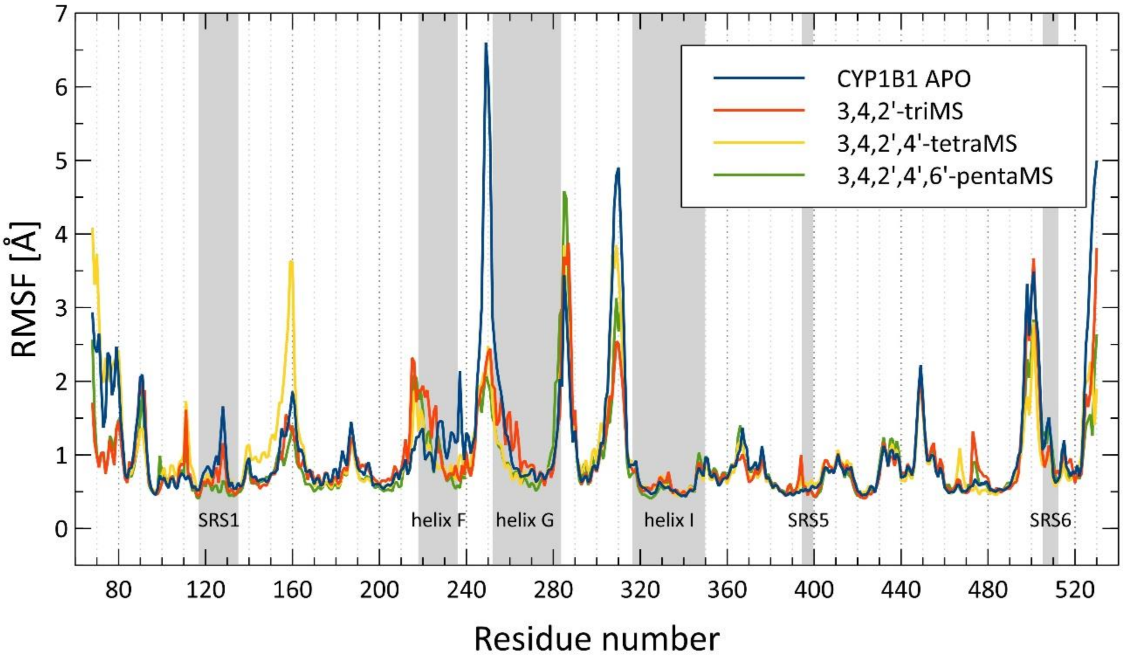

Figure S9. Root mean square fluctuations (RMSF) of protein backbone during MD simulations for CYP1B1 structures.
